# Zebrafish *arl6ip1* Is Required for Neural Crest Development during Embryogenesis

**DOI:** 10.1371/journal.pone.0032899

**Published:** 2012-03-09

**Authors:** Chi-Tang Tu, Tzu-Ching Yang, Hsing-Yen Huang, Huai-Jen Tsai

**Affiliations:** Institute of Molecular and Cellular Biology, National Taiwan University, Taipei, Taiwan; VIB & Katholieke Universiteit Leuven, Belgium

## Abstract

**Background:**

Although the embryonic expression pattern of ADP ribosylation factor-like 6 interacting protein 1 (Arl6ip1) has been reported, its function in neural crest development is unclear.

**Methods/Principal Findings:**

We found that knockdown of Arl6ip1 caused defective embryonic neural crest derivatives that were particularly severe in craniofacial cartilages. Expressions of the ectodermal patterning factors *msxb*, *dlx3b*, and *pax3* were normal, but the expressions of the neural crest specifier genes *foxd3*, *snai1b*, and *sox10* were greatly reduced. These findings suggest that *arl6ip1* is essential for specification of neural crest derivatives, but not neural crest induction. Furthermore, we revealed that the streams of *crestin-* and *sox10-*expressing neural crest cells, which migrate ventrally from neural tube into trunk, were disrupted in *arl6ip1* morphants. This migration defect was not only in the trunk neural crest, but also in the enteric tract where the vagal-derived neural crest cells failed to populate the enteric nervous system. We found that this migration defect was induced by dampened Shh signaling, which may have resulted from defective cilia. These data further suggested that *arl6ip1* is required for neural crest migration. Finally, by double-staining of TUNEL and *crestin*, we confirmed that the loss of neural crest cells could not be attributed to apoptosis.

**Conclusions/Significance:**

Therefore, we concluded that *arl6ip1* is required for neural crest migration and sublineage specification.

## Introduction

The neural crest (NC) contains pluripotent cells which are derived from the border of the neural and epidermal ectoderm and migrate through specific migration pathways in vertebrate embryos. NC cells contribute to the development of such cell types as facial cartilage and bones, neurons, pigment cells and glia of the peripheral nervous system, melanocytes of the skin, and smooth muscle of the heart. At the end of gastrulation, the NC arises at the border, which is also known as the neural fold, and flanks the neural plate bilaterally. During neurulation, the neural plate bends and folds into the neural tube and brings the neural folds together at the dorsal midline. During the folding process, NC precursors are contained within the neural fold. Subsequently, these cells undergo an epithelial-mesenchymal transition, which allows them to delaminate from the neuroepithelium and migrate away from the neural tube to various sites of the embryo [1], [2]. NC cells from the midbrain and hindbrain regions migrate throughout the head and contribute to most of the cartilage and bones of the head, as well as neurons of cranial ganglia. The caudal hindbrain zone of the NC, also known as the vagal crest, contributes to the heart and the enteric nervous system. The trunk NC cells give rise to sensory and sympathetic ganglia [3].

Because the NC possesses the ability to generate multiple cell lineages, any abnormal development of the NC has serious repercussions for many different organs. These pathologies, known as neurocristopathies, include conditions such as Waardenburg–Shah syndrome (hypopigmentation and aganglionic megacolon), frontonasal dysplasia (multiple craniofacial defects), and DiGeorge syndrome (craniofacial and heart defects). Therefore, discovering the mechanisms of NC formation and diversification represents an important step in understanding the basis of these pathologies [4].

NC development describes the progression of a NC cell over time from the initial commitment of the cell to its specific fate to its fully differentiated function. In this paper, we are particularly concerned with NC induction and sublineage specification. NC induction refers to the induction of ectoderm cells to the NC lineage and the ability to segregate and migrate away from the neuroepithelium. In fact, studies of mouse, frog, chick and zebrafish NC development have implicated multiple signaling molecules, including BMPs, FGFs, and Wnt proteins, as well as retinoic acid and Notch, in this process [5], [6], [7], [8], [9], [10], [11], [12], [13], [14]. In addition, the pre-migratory NC cells are identifiable by the expression of NC specifier genes, including *slug*, *ap-2*, *foxd3*, *sox10* and *pax3* [7], [9], [15], [16], [17], [18], [19], [20]. However, it remains unclear whether the genes encoding the molecular signals that initiate the migration of NC cells through the embryo are the same as those that determine their ultimate fate. Thus, the specification of NC at the neural plate border and the processes involved in determining the fate of individual neural crest progenitors are questions that remain to be elucidated. Nonetheless, it is thought that the downstream effector genes resulting from the interaction between inductive signals and transcriptional regulators govern NC development.

Following this reasoning, ADP-ribosylation factor-like 6 (Arl6), which belongs to the small ADP ribosylation factor GTP-binding proteins, is a major regulator in intracellular traffic, and Arl6ip1 is an interacting protein of Arl6. In mouse, *arl6ip1* is expressed in all hematopoietic cell lineages and in the early myeloid progenitor cells. Arl6ip1 protein is also dominantly localized in intracytoplasmic membranes, suggesting that *arl6ip1* functions in protein transport, membrane trafficking, or cell signaling during hematopoietic maturation [21]. Lui *et al.* (2003) [22] demonstrated that ARMER, also named human Arl6ip1, is a novel ER integral membrane protein which plays a possible role in cell survival by protecting cells through inhibition of caspase-9 activity. Mutation of Arl6 causes Bardet-Biedl syndrome (BBS), a multisystemic disorder characterized by obesity, blindness, polydactyly, renal abnormalities, cognitive impairment and Hirschprung disease [23]. Interestingly, analysis of BBS morphants in zebrafish showed that aberrant NC migration underlies the craniofacial and enteric nervous system defects mirroring mammalian mutants [24], suggesting that BBS-associated genes might be involved in NC migration. Recently, Huang *et al.* (2009) [25] reported that *arl6ip1* is a maternal gene and is expressed ubiquitously through 24 hours post-fertilization (hpf). They also showed that knockdown of *arl6ip1* by injection of *arl6ip1*-specific antisense morpholino oligonucleotides (MO; *arl6ip1*-MO1) resulted in displaying shortened mandibles with loss of pharyngeal arches and reduced pigmentation, suggesting that the NC of *arl6ip1*-MO1-treated embryos may be defective. By Western blot analysis, Huang *et al.* (2009) [25] demonstrated that the protein level of Arl6ip1 is greatly reduced in the *arl6ip1*-MO1-injected embryos, indicating the specificity and reliability of *arl6ip1*-MO1 by loss-of-function studies. Taken together, this evidence led us to hypothesize that *arl6ip1* may play an important role in NC development. Therefore, we investigated the function of zebrafish *arl6ip1* during embryogenesis by means of MO to knock down *arl6ip1* mRNA translation. Knockdown of Arl6ip1 activity resulted in reduction, or even loss, of NC derivatives, including pharyngeal arches, cranial and trunk ganglia, enteric neurons and pigment cells. While the induction of NC remained unchanged, later specification of sublineages was reduced in the *arl6ip1*-MO1-injected embryos. This line of evidence further implicated that *arl6ip1* plays a role in the specification of NC derivatives. In addition, we also proved that the down-regulation of the pre-migratory NC markers *foxd3*, *sox10*, and *snai1b* could not be attributed to cell death. Rather, it is suspected that a decreased level of these genes at early somatic stage may cause major defects in the specification of NC derivatives at later stages of embryonic development. Moreover, we concluded that *arl6ip1* may act parallel to, or downstream of, *pax3* and *msxb*, but upstream of *foxd3*, *sox10* and *snai1b*. This suggests that Arl6ip1 is not only required for specification of NC derivatives, but migration, as well. Finally, we demonstrated that *shh* signaling pathway may be disrupted by defective cilia, leading to the perturbation of NC cell migration and left-right laterality. Thus, the etiology underlying various neurocristopathies, as noted above, including craniofacial dysmorphology and Hirschsprung's disease in BBS, could very likely arise from abnormalities in *arl6ip1* expression and function, as these ultimately impact NC development.

## Results

### Knockdown of *arl6ip1* resulted in ectoderm-derived defects of embryos

**Figure 1:**
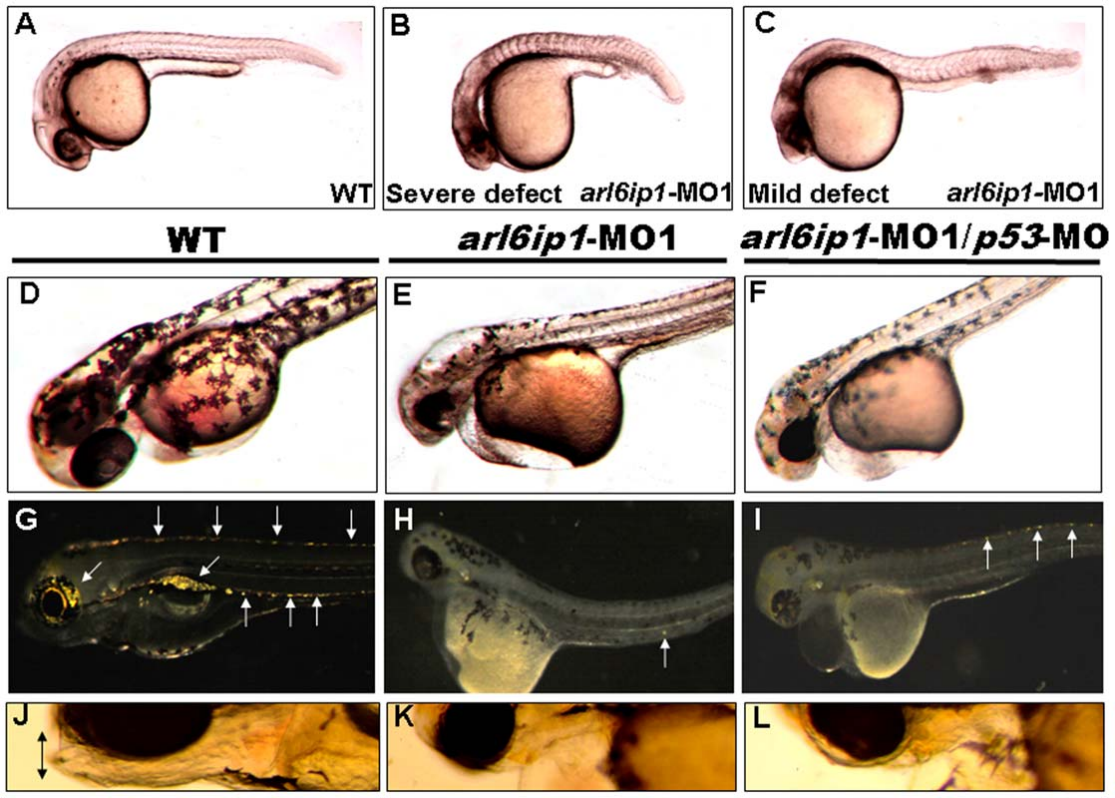
Embryos injected with *arl6ip1*-MO1 display abnormal development in ectoderm-derived tissues. Lateral views of embryos at 24 hpf (A–C), 48hpf (D–F) or at 96 hpf (G–L). (A) Wild-type (WT) embryos at 24 hpf displayed normal embryonic morphology. (B) Embryos injected with *arl6ip1*-MO1 exhibited foreshortened yolk extension and tail and small head with eye lesions. These embryos were defined as having severe defects. (C) Embryos with mild defects showed longer axis than embryos with severe defects. However, similar to embryos with severe defects, eye development was nearly equally compromised. (D) At 48hpf, WT embryos displayed plentiful melanophores. (E, F) Melanophores were highly reduced in *arl6ip1*-MO1 and *arl6ip1*-MO1*/p53*-MO-injected embryos. (G) Iridophores (arrows) were prominent in 4 dpf WT embryos. (H) Iridophores were almost absent in *arl6ip1*-MO1 embryos (only 1 residual cell was seen here in the ventral of the trunk) and (I) highly reduced in *arl6ip1*-MO1*/p53*-MO-injected embryos. (J) WT embryos showed well-developed head, jaw and melanophores. (K, L) *arl6ip1*-MO1 and *arl6ip1*-MO1*/p53-*MO-injected embryos displayed reduced head size and jaw (double arrows), but the head of *arl6ip1*-MO1*/p53-*MO embryos was bigger than *arl6ip1* morphants.

To investigate whether Arl6ip1 is required for early development, we injected embryos with *arl6ip1-*MO1 which specifically blocked the translation of *arl6ip1* mRNA. Many morphological defects were observed in the 4 ng *arl6ip1-*MO1-injected embryos, compared to the control-MO-injected embryos. For example, at 24 hpf, the yolk extension and tail were foreshortened, pericardial edema was present, lens and otic vesicles were absent or highly reduced in size, and the head became smaller (Figs. 1A–C). However, since apoptosis can be induced by MO-toxicity, *p53-*MO was reported by Robu *et al.* (2007) as an excellent tool to attenuate neural cell death, but with no effect on embryonic morphology [26]. Therefore, we co-injected *p53*-MO to prevent nonspecific defects caused by cell death. Compared to wild-type embryos, both *arl6ip1*-MO1 and *arl6ip1*-MO1 combined with *p53*-MO (*arl6ip1-*MO1/*p53*-MO) morphants showed reduced cell numbers and mismigration of melanophores at 48 hpf (Figs. 1D–F). At 96hpf, the iridophore pigment cells of *arl6ip1-*MO1 morphants, as well as *arl6ip1-*MO1/*p53*-MO-injected embryos, were much reduced in comparison to wild-type embryos (Figs. 1G–I, indicated by arrows).. The jaw morphology shown in wild-type embryos at 96 hpf (indicated by double arrowhead in Fig. 1J) did not form in *arl6ip1-*MO1-injected and *arl6ip1-*MO1/*p53*-MO-injected embryos (Figs. 1K and L). The occurrence rates of reduced iridophore cells and melanophores in the 4 ng *arl6ip1*-MO1-injected embryos and in the 4 ng *arl6ip1*-MO1/6ng *p53*-MO- injected embryos were 84.5% (93/110 ) and 79.5% (128/161), respectively. The head size and trunk extension were also recovered in *arl6ip1-*MO1/*p53*-MO-injected embryos at 24 hpf (data not shown). Since the off-targeting effect was inhibited by *p53*-MO, no NC derivatives could be rescued in the *arl6ip1*-MO1/*p53*-MO-injected embryos. We also injected 8 ng of *arl6ip1*-MO2 into embryos, and the resulting defects phenocopied those of *arl6ip1-*MO1-injected embryos (data not shown) [25]. These findings suggested that knockdown of Arl6ip1 function causes defects related to NC degeneration.

The occurrence rate of defects induced by injection of *arl6ip1-*MO1 was dose-dependent (Table 1). We observed that higher concentrations of injected *arl6ip1-*MO1 resulted in a correspondingly higher rate of defects. However, when 4 ng *arl6ip1*-MO1 were co-injected with 150 and 200 pg of a wobble *arl6ip1* mRNA in embryos, the occurrence rates of mild defects were observed to decrease from 48.4% to 32.1% and 17.6%, respectively, while the occurrence rates of the wild-type-like phenotype increased from 16.1% to 56% and 77%, respectively (Table 1). Furthermore, co-injection of 4 ng *arl6ip1*-MO1/*p53*-MO with 75 and 150 pg of a wobble *arl6ip1* mRNA in embryos decreased the occurrence rates of mild defects from 74% to 34.5% and 17.5%, respectively, but raised the occurrence rates of wild-type-like phenotype from 14.4% to 60.2% and 80.1%, respectively (Table 1, Fig. S1K). In addition, we co-injected a wobble *arl6ip1-gfp* mRNA with *arl6ip1*-MO1, and the Arl6ip1-GFP fusion protein was detected in embryos (Figs. S1B and F), indicating that the injected *arl6ip1*-MO1 failed to inhibit the translation of the introduced wobble *arl6ip1-gfp* mRNA. Furthermore, either co-injection of *arl6ip1*-MO1 or *arl6ip1-*MO1/*p53*-MO with a wobble *arl6ip1* mRNA rescued the defective phenotypes in embryos induced by *arl6ip1*-MO1 (Figs. S1B and C, Table 1). To further confirm whether phenotypes of these morphants were specifically induced by the absence of Arl6ip1 function, we constructed a plasmid of pCS2-Arl6ip1-GFP, in which the targeting sequence of *arl6ip1*-MO1 was fused with the GFP cDNA. The GFP was expressed in the embryos injected with 4 ng *arl6ip1*-MO1 together with 100 pg *GFP* mRNA (*n* = 112), whereas the expression of GFP was totally absent in the embryos injected with 4 ng *arl6ip1*-MO1 together with 100 pg *arl6ip1*-MO1-target- *gfp* RNA synthesized from pCS2-Arl6ip1-GFP (*n* = 115) (Figs. S1D and H). This line of evidence indicated that *arl6ip1*-MO1 was capable of specifically blocking the translation of *arl6ip1* mRNA and that the defects induced by *arl6ip1*-MO1 were specific and could be rescued by wobble *arl6ip1* mRNA.

**Table 1:** Injection of wobble *arl6ip1* mRNA enables embryos to rescue the defects induced by injection of *arl6ip1*-MO1/*p53*-MO.

*arl6ip1*-MO1-injected concentration	*p53*-MO-injected concentration	Defects (%)		Wild-type-like
		Severe	Mild	
2 ng	—	14.3(12/84)	42.9(36/84)	42.9(36/84)
4 ng	—	35.5(55/155)	48.4(75/155)	16.1(25/155)
6 ng	—	41.1(46/112)	55.4(62/112)	3.6(4/112)
4 ng+150 pg *arl6ip1* mRNA	—	11.9(13/109)	32.1(35/109)	56.0(61/109)
4 ng+200 pg *arl6ip1* mRNA	—	5.4(8/148)	17.6(26/148)	77.0(114/148)
—	6 ng	—	1.9 (2/105)	98.1 (103/105)
—	8 ng	—	4.2(5/118)	95.8(113/118)
4 ng	6 ng	11.5(12/104)	74.0(77/104)	14.4(15/104)
4 ng+75 pg *arl6ip1* mRNA	6 ng	5.3(6/113)	34.5(39/113)	60.2(68/113)
4 ng+150 pg *arl6ip1* mRNA	6 ng	2.4(4/166)	17.5(29/166)	80.1(133/166)
4 ng+0.2 pg *foxd3* mRNA	6 ng	13.6(12/88)	61.2 (54/88)	25.0 (22/88)
4 ng+0.4 pg *foxd3* mRNA	6 ng	16.2(12/74)	51.4(38/74)	32.4(24/74)

The morphological defects of embryos were observed at 36 hpf. Severe defects are defined as those where the brain did not form sulcus and gyrus in the head and where the axis appeared as a curved shape in the trunk of embryos. Mild defects are defined as those where the embryonic axis was longer than observed in embryos with severe defects, but where head defects were just as severe. The *arl6ip1* mRNA used in this study was synthesized as a wobble form.

### Pharyngeal arch development is abnormal in *arl6ip1* morphants

**Figure 2:**
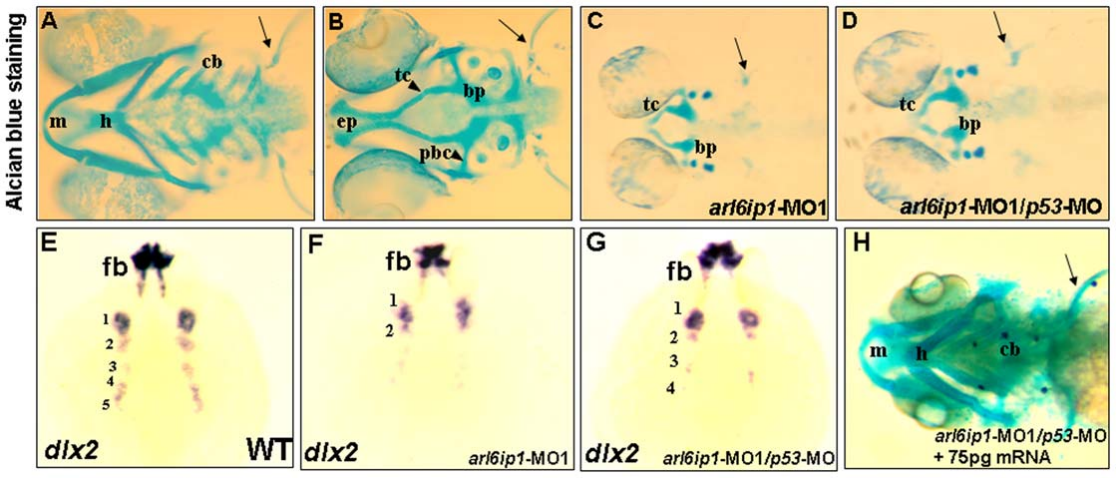
Craniofacial cartilage defects in the *arl6ip1* morphants. Ventral views (A, C, D, H) or dorsal view (B) of 4-dpf embryos stained with Alcian blue to reveal craniofacial cartilage. Either pectoral fin (pf) or cartilage derived from mandibular (1st arch, m), hyoid (2nd arch, h), and ceratobranchial (3rd–7th arches, cb) arches was observed in control embryos (A). Dorsal views of anterior neurocranial cartilage in wild-type embryos showed paired trabeculae (tc) and an ethmoid plate (ep) (B). However, craniofacial cartilage and pectoral fin were absent in the *arl6ip1-*deficient (C) and *arl6ip1*-MO1/*p53*-MO-injected embryos (D). In the anterior neurocranial cartilage, trabeculae were severely reduced in both of these embryos, and the ethmoid plate was absent (C, D). *dlx2* was expressed in migratory neural crest precursors of pharyngeal arch, as shown in dorsal views at 36 hpf in wild-type (E). Both *arl6ip1*-MO1-injected (F) and *arl6ip1*-MO1/*p53*-MO-injected embryos (G) had reduced *dlx2* expression, and the reduction of *dlx2* was prominent in posterior arches at 24 hpf. In addition, *dlx2* was expressed normally in the forebrain (E, F, G). The severe chondrocranium and pectoral fin defects in *arl6ip1*-MO1/*p53*-MO-injected embryos were rescued by injection of 75 pg wobble *arl6ip1* mRNA (H). bp, basal plate; pbc, posterior basic capsular commiss. Arrows, pectoral fin.

When Arl6ip1 was knocked down, the mandible was reduced, as mentioned above. The majority of the head skeleton is derived from the cranial neural crest cells which migrate to the branchial arches and frontonasal process. Therefore, we speculate that the shortened mandible of *arl6ip1* morphants may have resulted from defective craniofacial cartilages. To test this hypothesis, we performed Alcian blue staining to observe the craniofacial cartilage of the 4-dpf embryos. The craniofacial cartilages, such as mandibular (1st arch), hyoid (2nd arch) and ceratobranchial (3rd–7th arches), which are all derived from three streams of cranial NC [27], were all clearly shown (Fig. 2A). In contrast, no cartilages for any of the arches were apparent in either the *arl6ip1*-MO1-injected embryos or *arl6ip1-*MO1/*p53*-MO-injected embryos (Figs. 2C and 2D). The occurrence rate of neurocranium defects, either in the 4 ng *arl6ip1*-MO1-injected embryos or in the 4 ng *arl6ip1*-MO1/6 ng *p53*-MO- injected embryos, was 100% (133/133 in *arl6ip1*-MO1 embryos; 146/146 in *arl6ip1*- MO1/*p53*-MO embryos). Moreover, severe shortening of trabeculae and complete loss of ethmoid plate (Fig. 2C), which are from anterior neurocranial cartilage, were also observed in the *arl6ip1*-MO1-injected embryos. However, wild-type embryos displayed a well-organized anterior neurocranial cartilage (Fig. 2B). It should be noted that both of these cartilages were derived from NC for the development of neurocranium. Finally, *arl6ip1*-MO1/*p53*-MO-injected embryos exhibited the same craniofacial phenotypes as the *arl6ip1*-MO1-injected embryos (Fig. 2D), except for the larger head size. Furthermore, the severe craniofacial defect, which is shown in Figures 2D, could be rescued in wobble *arl6ip1* mRNA-injected embryos (Fig. 2H). Since the co-injection of *p53*-MO was used to rule out nonspecific effect by MO-toxicity [26], we are satisfied that these *arl6ip1*-MO1/*p53*-MO co-injection data indicated that the complete loss of both pharyngeal arches and ethmoid plate with shortened trabeculae could not be attributed to either cell death or the nonspecific effects of *arl6ip1*-MO1. Instead, Arl6ip1 was clearly demonstrated to function in NC derivative arches.

Schilling and Kimmel (1997) [27] demonstrated that *dlx2* is required for jaw development and is expressed by NC cells, which contribute to the pharyngeal arches, as well as by migratory arch-associated NC cells. While we found a marked increase of *dlx2* expression in the migratory NC pharyngeal arch precursors in wild-type zebrafish embryos (Fig. 2E), we also found that *dlx2* was markedly reduced in the migratory NC cells destined for the posterior arches of the *arl6ip1* and *arl6ip1*/*p53* morphants (Figs. 2F and G), suggesting that reduced *dlx2* expression in the morphants is responsible for the defective craniofacial cartilages. Furthermore, the expression pattern of *dlx2* in the forebrain of *arl6ip1*-MO1-injected embryos was similar to that of wild-type embryos, indicating that the differentiation in the forebrain was normal.

### NC derivatives are reduced in *arl6ip1-*deficient embryos

**Figure 3:**
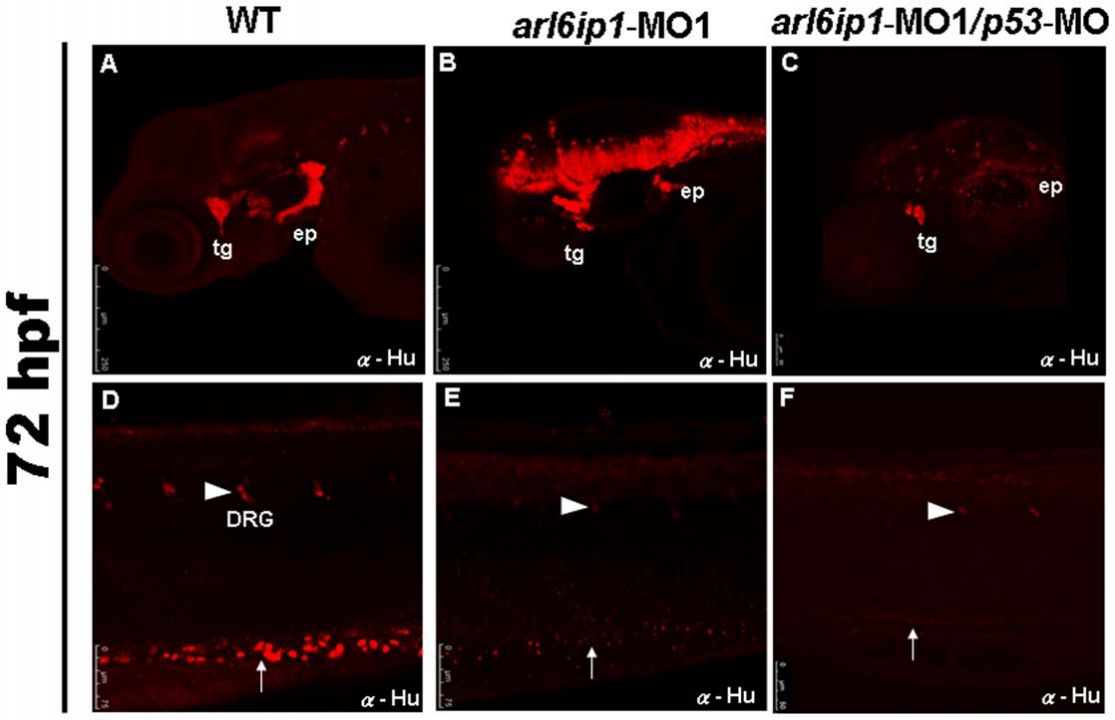
Neural crest derivatives are reduced in *arl6ip1*-deficient embryos. Lateral views (A–F) of 72 hpf embryos processed for anti-Hu immunofluorescence assaying (IFA) to reveal the derivatives of cranial neural crest cells by confocal micrographs. In control embryos (A, D), anti-Hu IFA-labeled trigeminal ganglia (tg), epibranchial ganglia (ep), dorsal root ganglia (drg) (arrowhead), enteric neurons (arrow) all appeared to be reduced either in *arl6ip1* knockdown embryos (B, E) or *arl6ip1*-MO1*/p53*-MO-injected embryos (C, F).

The occurrence of defective cartilages in the *arl6ip1*-MO1-injected embryos strongly suggested that *arl6ip1* may play a role in NC development. To test this hypothesis, we used an anti-Hu immunofluorescence assay to examine the cranial ganglia surrounding the otic vesicles or the dorsal root ganglia and enteric neurons in the trunk, since these ganglia have NC origin. Results showed a severe reduction of neurons in epibranchial ganglia, dorsal root ganglia and enteric ganglia, and a mild reduction of neurons in trigeminal ganglia, either in the *arl6ip1* or *arl6ip1*/*p53* morphants (Figs. 3B, C, E and F). The occurrence rates of cranial ganglia, dorsal root ganglia and enteric neuron defects in the 4 ng *arl6ip1*-MO1-injected embryos were 68.3% (71/104), 72.5% (132/182) and 73.8% (48/65), respectively. These data indicated that cranial, vagal and trunk neurons, which are derived from the NC, were defective in the *arl6ip1*-MO1-injected embryos. We also detected a large number of Hu-positive cells in the head of *arl6ip1*-MO1-injected embryos (Fig. 3B). To explain this observation, we suggest that antibody penetration into the brain of morphants is more efficient, making brain neurons more visible.

### Arl6ip1 is required for the specification of NC sublineages

**Figure 4:**
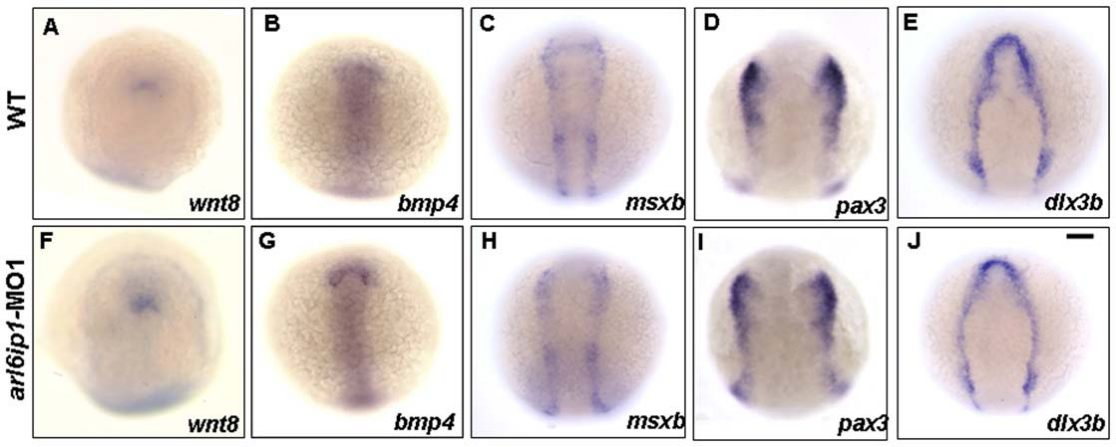
Expressions of ectodermal patterning factors appear normal in *arl6ip1*-MO1-injected embryos. Dorsal views of wild-type (A–E) and *arl6ip1* morphant (F–J) embryos at the 3-somite stage. Anterior to the top. The neural ectodermal patterning factors of *wnt8* (A, F), *bmp4* (B, G), *msxb* (C, H), *dlx3b* (D, I), and *pax3* (E, J). Normal expressions of these genes reveal that ectodermal patterning factors are not affected in *arl6ip1* morphants, suggesting that *Arl6ip1* does not have a role in ectodermal patterning. Scale bar: 100 µm.

**Figure 5:**
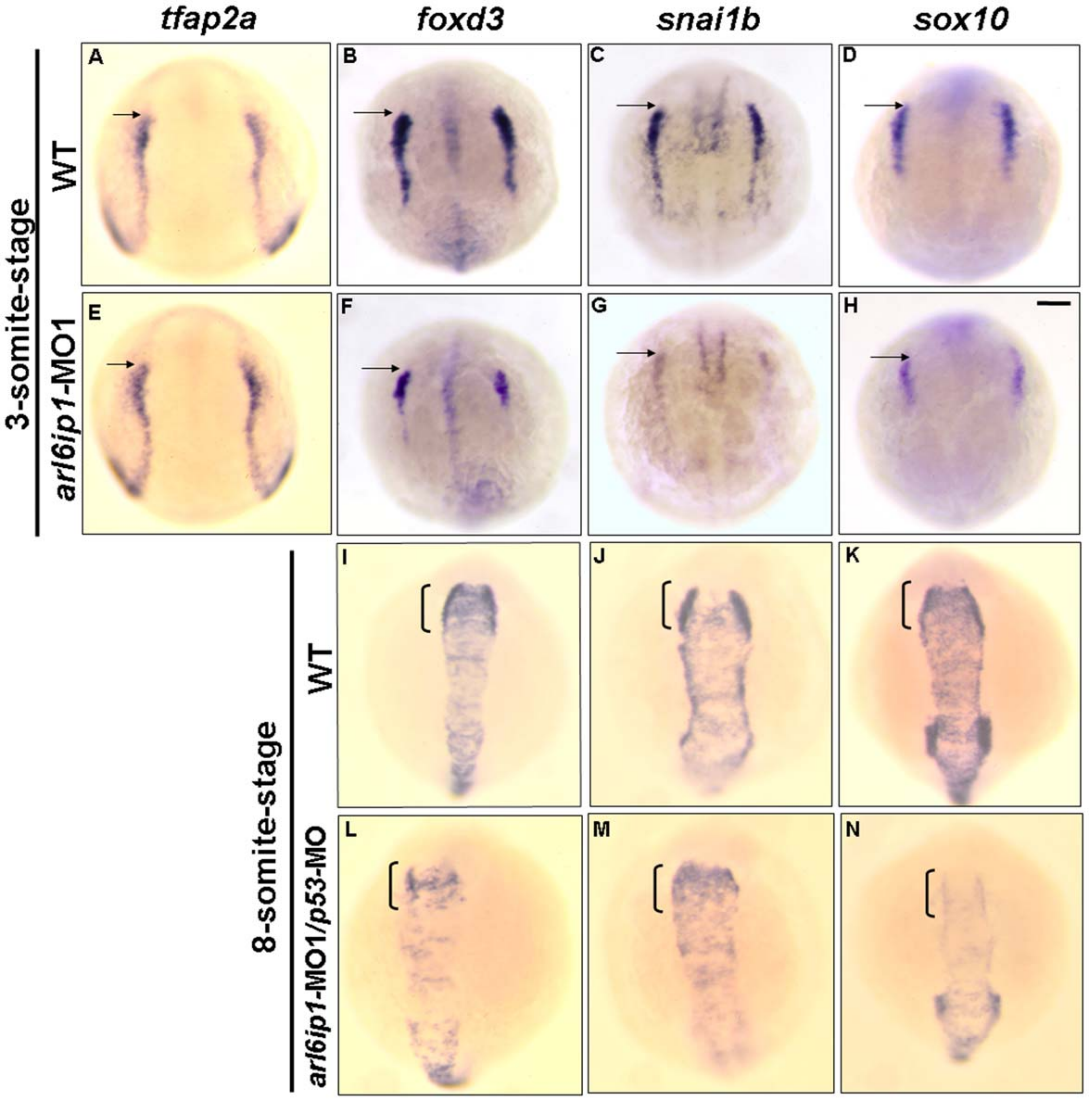
The markers of pre-migratory neural crest cells are down-regulated in *arl6ip1*-MO1-injected embryos. Dorsal views of 3-somite-stage wild-type (A, B, C, D) and *arl6ip1* knockdown embryos (E, F, G, H), or 8-somite-stage wild-type (I, J, K,) and *arl6ip1* knockdown embryos (L, M, N) were shown to reveal the indicated markers. Neural plate border was indicated with the expression of *tfap2α* (A, E), *foxd3* (B, F), *snai1b* (C, G) and *sox10* (D, H). Decreased expression of these markers, except *tfap2α*, was evident and predominant in *arl6ip1* morphants. Arrows indicate position of midbrain-hindbrain boundary. (I–K) At the 8-somite stage, these markers were expressed predominantly in early migrating cranial neural crest cells (indicated by brackets). (L–N) These genes were reduced in embryos co-injected with 4 ng *arl6ip1*-MO1/6 ng *p53*-MO, especially in *foxd3* and *snai1b* expression.

To study the function of *arl6ip1* during NC development, we first detected the expressions of the ectodermal patterning factors *wnt8*, *bmp4*, *pax3*, and *msxb*. The ectodermal patterning factors were all normally expressed in the *arl6ip1*-MO1- injected embryos (Figs. 4A and F; 4B and G; 4C and H; 4D and I). In addition, the expression of the pre-placodal marker *dlx3b* was also normal in the *arl6ip1*-MO1- injected embryos (Figs. 4E and J, n = 64). Furthermore, since Trainor and Krumlauf (2000) [28] reported that segmentation and patterning of the hindbrain are necessary for cranial NC development; we also examined the expressions of two hindbrain patterning markers, *fgf3* and *fgf8*. Results showed that *fgf3* and *fgf8* were also expressed normally in the hindbrain of *arl6ip1* morphants (Figs. S2A–D, n = 87) and that 83.2% of morphants exhibited a normal hindbrain segmentation at 24 hpf (Figs. S2E–F, n = 101). These findings suggested that the induction of both early NC progenitors and placodal region cells results in normal development of *arl6ip1* morphants. The early patterning and later segmentation of the hindbrain in the *arl6ip1*-MO1-injected embryos were also similar to those of wild-type. Thus, even though *arl6ip1* may not function in hindbrain development, it is expressed ubiquitously until 24 hpf, and knockdown of Arl6ip1 did cause small head morphology. This line of evidence suggests that neural crest defects in *arl6ip1*-MO1 embryos did not result from abnormal head development. On the other hand, when we observed the expressions of pre-migratory NC markers in the progenitors of the neural plate border, such as *tfap2α*, *foxd3*, *snai1b*, and *sox10*, we found that the expressions of *foxd3*, *snai1b* and *sox10* were greatly reduced in the *arl6ip1* morphants at 3-somite (Figs. 5B and F; 5C and G; 5D and H) through 8-somite stages (Figs. 5I and L; 5J and M; 5K and N). Meanwhile, *tfap2a* expression in NC progenitors was present in both wild-type and *arl6ip1*-MO1 embryos (Figs. 5A and E). Double-staining of *sox2* (labeling neural plate) and *dlx3b* (labeling the pre-placodal region) exhibited a patch of unlabeled cells between these two expression domains which was presumed to be the NC. Thus, since the induction of presumptive NC was normal in *arl6ip1*-MO1 embryos (Fig. S3), we concluded that NC induction occurred normally in *arl6ip1* morphants. The occurrence rates of down-regulation of *foxd3*, *snai1b* and *sox10* in the *arl6ip1*-MO1-injected embryos were 75.6% (31/41), 82.2% (37/45) and 66.7% (30/45), respectively. Again, since down-regulation of these genes could have resulted from early cell death by the off-targeting effect of MO, we injected 6 ng *p53-*MO together with 4 ng *arl6ip1*-MO1 to inhibit apoptosis and nonspecific effects. We found that the level of cell death was dramatically reduced in the embryos injected with *p53-*MO. In addition, Cole and Ross (2001) [29] have detected the temporal and spatial distribution of apoptotic cells during normal development of the zebrafish embryo from 12 to 96 hpf. According to their data, only a few apoptotic cells could be seen in the optic vesicle at 12hpf, and no apoptosis was observed in the pre-migratory neural crest cell region. Robu *et al.* (2007) [26] also demonstrated that about 15–20% of MO used in zebrafish show off-targeting effects, as represented by a signature neural death peaking at the end of segmentation (1 dpf). They also showed that the neural cell death induced by MO was observed as early as 14 hpf. Moreover, Ekker and Larson (2001) [30] indicated that the onset of cell death induced by MO technology was 14 hpf. In our data, we detected pre-migratory neural crest markers at the 3-somite stage (about 10.33 hpf), even earlier than 12 hpf; consequently, we did not observe off-targeting effects at the 3-somite stage. On the other hand, when *p53* was inhibited, cell survival could not rescue expressions of *foxd3*, *snai1b* and *sox10* at the 8-somite stage (Figs. 5L–N). Therefore, we concluded that the down-regulation of these gene markers in existing NC cells was caused by defects in NC derivate specification, not cell death. Furthermore, such defects subsequently resulted in the reduction of the cell population, especially in pharyngeal arches of cranial NC sublineages. Finally, we found that the reduced expression of *foxd3* induced by *arl6ip1*-MO1 could be restored by injection of wobble *arl6ip1* mRNA in *arl6ip1*- MO1-injected embryos (Figs. S4A–C). These findings implicated that Arl6ip1 plays a key function in specification of NC sublineages, but not induction.

### Trunk NC migration is defective, and cranial NC is reduced in the *arl6ip1* morphants, as co-tested with *myod* staining

**Figure 6:**
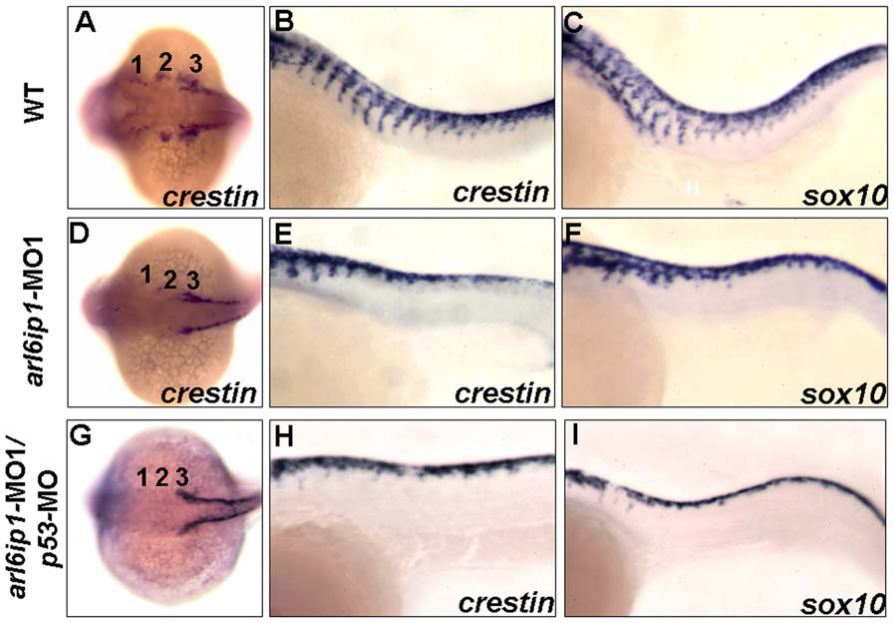
Abnormal cell migration in *arl6ip1*-MO1-injected embryos. Wild-type (A–C), *arl6ip1-*MO1-injected (D–F) and *arl6ip1*-MO1/*p53*-MO-injected (G–I) embryos were shown under dorsal views at 20-hpf (A, D, G) and under lateral views at 26-hpf (B, C, E, F, H, I), anterior to the left. *Crestin* expression revealed the migration of the three cranial neural crest streams in the wild-type embryos at 20 hpf (A). In *arl6ip1* and *arl6ip1*/*p53* knockdown embryos, *crestin* expression in the cranial crest was reduced in the 3rd neural crest stream and almost absent in the 1st and 2nd stream (D, G). At 26 hpf, the trunk migratory neural crest cells in wild-type embryos, as labeled by *crestin* and *sox10*, gradually migrated to ventral (B, C). However, the *sox10-* and *crestin-*expressing cells in *arl6ip1* knockdown and *arl6ip1*-MO1/*p53*-MO-injected embryos failed to migrate ventrally (E, F, H, I).

In wild-type zebrafish embryos, the migration of cranial NC has three distinct streams: migration from rhombomeres (r) r2, r4 and r6 to mandibular (1st arch), hyoid (2nd arch) and ceratobranchial (3rd–7th arches), respectively. We were able to detect these migratory NC streams by *crestin* probe (Fig. 6A). In the *arl6ip1*-MO1-injected embryos at 24 hpf, the *crestin* expression in the cranial crest was reduced in the 3rd NC stream, and *crestin* was almost absent in the 1st and 2nd stream, as shown in Figure 6D. The reduced expression of *crestin* in cranial NC cells resulted in defective craniofacial cartilages, which also was proved by previous data of *dlx2*, as shown in Figure 2. The occurrence rate of reduced defects in the *crestin*-labeled cranial NC was 73.4% (47/64). However, the trunk NC cells in the *arl6ip1* morphants appeared to have defects in NC migration. Because severe trunk extension and somite defects could indirectly affect trunk neural development, such as cell migration, we used *arl6ip1* and *arl6ip1/p53* morphants with only mild defects impeding normal trunk development to analyze the migration of trunk NC. Compared to wild-type embryos, there was a significant delay in the onset of migration of the *arl6ip1* morphants, which had only a small number of *ctn*-expressing cells migrating within the most anterior somites (Figs. 6B and E). Similarly, the *sox10* gene, which is also expressed in migrating trunk NC cells [31], showed a delay in NC migration in the *arl6ip1*-MO1-injected embryos at 24 hpf (Figs. 6C and F). Compared to wild-type embryos, the cell numbers in trunk NC of *arl6ip1*-MO1-injected embryos were normal, but these cells could not migrate. The occurrence rates of migration defects in the *sox10*- and *crestin*-labeled trunk NC were 72.7% (40/55) and 75% (45/60), respectively. Co-injection of *arl6ip1*-MO1 with *p53*-MO in embryos showed the same migration defects as the *arl6ip1*-MO1-injected embryos (Figs. 6H and I). To quantify the number of streams of *crestin*-positive NC cells which migrate through the trunk, we observed that the inhibition of migrating streams of NC cells from the dorsal neural tube was exhibited in the *arl6ip1* morphants (Fig. S4G). Again, injection of a wobble *arl6ip1* mRNA rescued embryos from these defects (Figs. S4D–F and S4G). When we traced the expressions of *crestin* and *sox10* at 30 hpf, we found that the streams of *sox10*- and *crestin*-labeled cells in the *arl6ip1* morphants still could not migrate normally when compared to the wild-type embryos (data not shown). This evidence revealed that trunk NC cells remained localized between the dorsal surface of the neural keel and overlying ectoderm in the *arl6ip1*-MO1-injected embryos (Figs. 6E and F). In summary, the defects shown in dorsal root ganglia clearly resulted from the inhibition of trunk NC migration.

**Figure 7:**
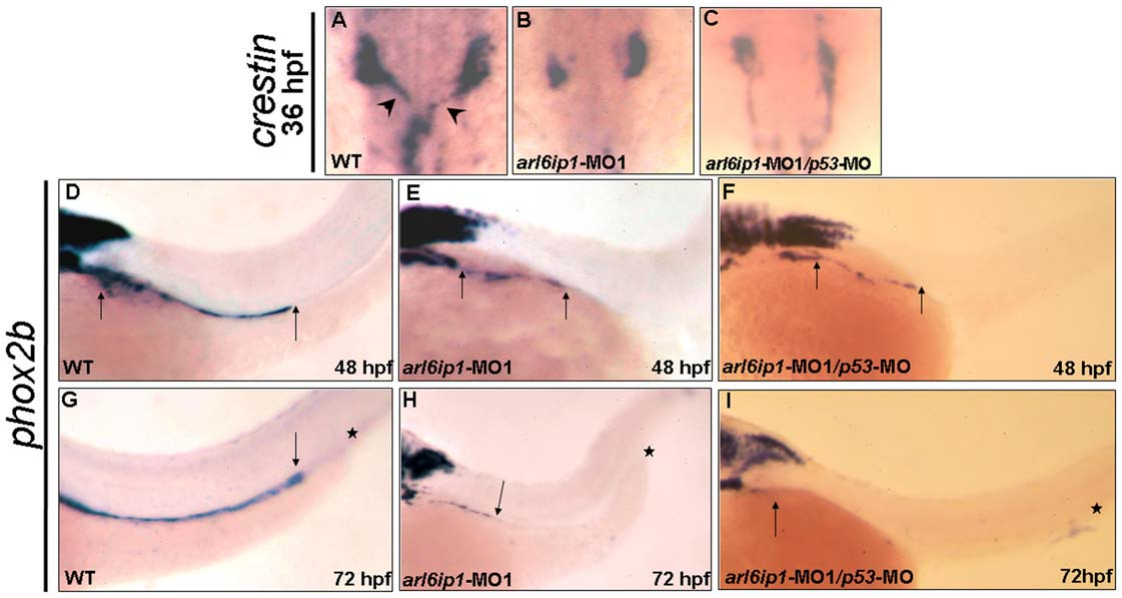
Enteric nervous system of *arl6ip1* morphants is degenerated. Wild-type (WT) embryos (A, D, G), *arl6ip1*-MO1-injected embryos (B, E, H), and *arl6ip1*-MO1/*p53*-MO-injected embryos (C, F, I) were observed under dorsal view at 36-hpf (A–C) and under lateral view at 48 hpf (D, E, F) and 72 hpf (G, H, I). Neural crest cells (NCCs), which were labeled by *crestin* probe (arrowheads in A), of WT embryos exited the vagal region and migrated to the enteric region. However, the *crestin*-labeled NCCs of *arl6ip1*-MO1-injected and *arl6ip1*-MO1/*p53*-MO-injected embryos remained in the vagal region (B, C). Compared to WT embryos, either the *arl6ip1*-MO1-injected embryos or *arl6ip1*-MO1/*p53*-MO-injected embryos exhibited shortened enteric neurons and delayed migration (D vs. E; D vs. F; distance between two arrows). Additionally, the *phox2b*-positive enteric neurons were distributed throughout the entire gut to the anus (G). Arrows indicate the most posterior region of migration, and the asterisks represent the anus. The enteric neurons of *arl6ip1*-MO1-injected embryos and *arl6ip1*-MO1/*p53*-MO-injected embryos all failed to reach as far as the anus (H, I).

To further understand why enteric neurons were absent in the *arl6ip1-*deficient embryos, as shown in Figure 3D, we examined vagal NC migration. We observed embryos at 36 hpf and found that the *crestin*-labeled NC cells of wild-type embryos migrated out of the vagal region (Fig. 7A). However, the *crestin*-labeled NC cells of *arl6ip1*-MO1-injected and *arl6ip1*-MO1*/p53*-MO-injected embryos did not migrate normally and showed decreased progenitor cell numbers when compared to the wild-type (Figs. 7B and C). Additionally, we observed that the enteric nervous system (ENS) precursors labeled by *phox2b* were distributed along the entire length of intestine from 48 to 72 hpf in wild-type embryos (Figs. 7D and G). In contrast, these *phox2b*-positive ENS precursors only migrated to the anterior part of the intestine in *arl6ip1* morphants and *arl6ip1/p53* morphants (Figs. 7E and F; 7H and I). The occurrence rate of migration defects in the *phox2b*-labeled enteric neurons was 88.7% (63/71). We suggested that this migratory delay and partial loss of enteric precursors would affect normal differentiation of enteric neurons and might lead to loss of the ENS.

### Disruption of zebrafish Arl6ip1 function resulted in cilia defects and dampened Hedgehog signaling

During migration, Shh directs migration and patterning of NC cells. Tobin *et al.* (2008) [24] indicated that knockdown of BBS disrupted the Sonic Hedgehog (Shh) signaling pathway and affected NC cell migration. Therefore, we investigated Shh signaling in *arl6ip1* morphants. The results showed that *shh* expression was unaltered in *arl6ip1*-MO1*/p53*-MO-injected embryos (Fig. S5B); however, *pax6*, normally repressed by Shh signaling, was up-regulated and ectopically expressed throughout the neural tube in *arl6ip1*-MO1*/p53*-MO-injected embryos (Fig. S5D). The occurrence rate of overexpressed *pax6* in the in the neural tube was 78.4% (69/88). We therefore concluded that the Shh signaling pathway might be perturbed in *arl6ip1*-MO1*/p53*-MO-injected embryos. In addition, Shh signaling reveals a cell autonomous requirement for Hh signal transduction and is directly required for normal development of DRG neurons in zebrafish [32]. Thus, the defects shown in dorsal root ganglia (Fig. 3) clearly resulted from disrupted Shh signaling pathway. Previous study demonstrated that cilia are required for normal Hh signaling [33]. Furthermore, overexpression of GDP- or GTP-locked variants of ARL6/BBS3 in a ciliated cell line, namely retinal pigmented epithelial cells (hTERT-RPE), influences primary cilium length and abundance [34]. Therefore, we additionally determined whether cilia of *arl6ip1*-MO1*/p53*-MO-injected embryos were normal. Kupffer's vesicle (KV) contains abundant, evident and aggressive cilia in zebrafish. By confocal immunostaining of acetylated tubulin in 8-somite stage embryos, apical cilia in the KV were shortened and reduced in number in *arl6ip1*-MO1*/p53*-MO-injected embryos compared with the wild-type embryos (Figs. S6I and J). The occurrence rate of reduced KV was 51.6% (49/95). Since zebrafish KV plays a role in left-right patterning [35], we tested whether cilia defect in KV resulted in laterality defects by assaying expression of two conserved left-right genes, *southpaw* (*spaw*) and *pitx2*. Significantly, *arl6ip1*-MO1*/p53*-MO-injected embryos showed randomized expression of *spaw* and *pitx2* (Figs. S6A–H and S6K). Finally, following the method reported by Amack and Yost (2004) [35], we injected fluorescence-tagged *arl6ip1*-MO1 into embryos between 2 to 4 hpf and selected embryos whose MOs had accumulated into dorsal forerunner cells (DFC) to determine if Arl6ip1 was required for KV morphogenesis. The occurrence rate of mid-blastula-injected MOs, which were incorporated into DFC, was 42% (n = 435). The results showed that DFC of *arl6ip1*-MO1-injected embryos displayed reduced KV (65%, n = 133) and even failed to form KV (23%, n = 133). Thus, cilia were disorganized in the KV of DFC of *arl6ip1*-MO1-injected embryos (Fig. S7). Taken together, we demonstrated that Arl6ip1 is required for KV morphogenesis and that *shh* signaling may be perturbed by defective cilia, in turn, leading to the perturbation of NC cells migration and left-right laterality.

### Loss of Arl6ip1 function causes cell death in embryos, but not in NC at later stage

**Figure 8:**
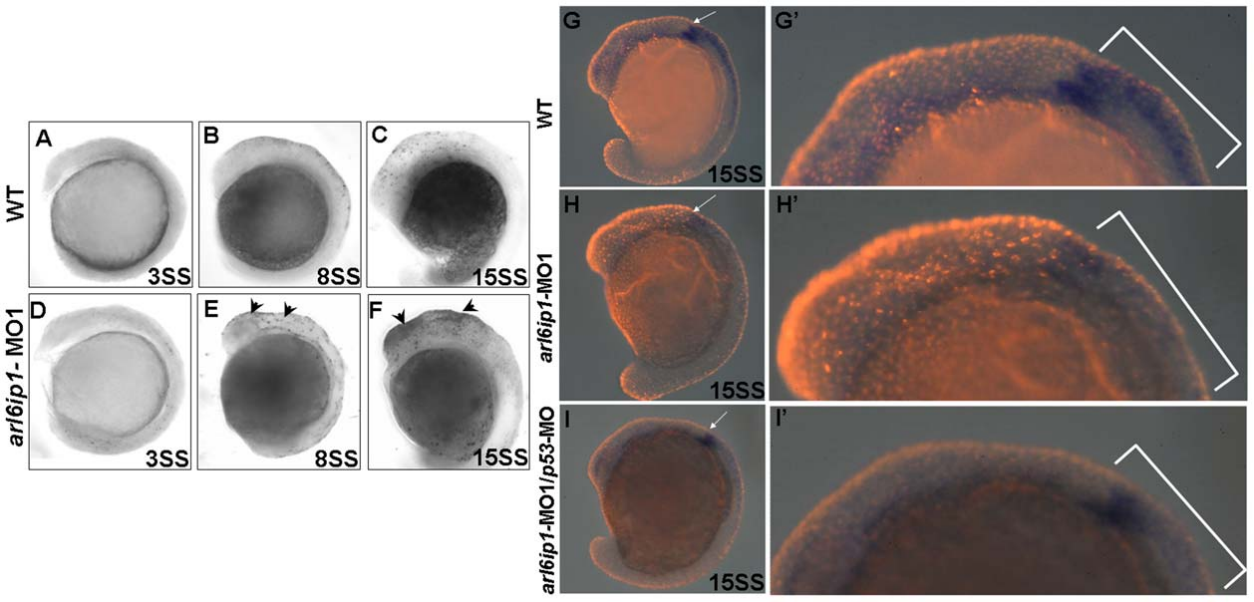
Cell death does not cause the loss of neural crest cells. Lateral views of TUNEL-labeled wild-type (WT) embryos (A–C) or *arl6ip1*-MO1-injected embryos (D–F) at 3-somite stage (A, D), 8-somite stage (B, E), and 15-somite stage (C, F), respectively. (A, D) There were no TUNEL-positive cells in WT and *arl6ip1*-MO1 embryos. (B, C, E, F) Compared to WT embryos, *arl6ip*-deficient embryos displayed a significant increase of TUNEL-positive cells in the dorsal region of embryos at the 8- and 15-somite stages (indicated by arrowheads) (B and C vs. E and F). (G, H, I) Double-labeling analysis with *crestin* (dark blue) and TUNEL (red fluorescent) showed that only limited cell death occurred at the expression region of *crestin*. (G′, H′, I′) The views of the embryos shown in panels G′, H′ and I′ were at higher magnification.

In *arl6ip1*-deficient embryos, pharyngeal arches, dorsal root ganglia and enteric neurons at the later stages of embryonic development were severely reduced or absent. The loss of these cells during development might result from cell death. To investigate the role of Arl6ip1 in NC survival, TUNEL assay was performed to examine cell death. At the 3-somite stage, there were no significant TUNEL signal differences in cell death between wild-type and *arl6ip1*-MO1-injected embryos (Figs. 8A and D). However, we detected a great increase of TUNEL-positive cells around the dorsal ectoderm in *arl6ip1*-MO1-injected embryos from the 3- to 15-somite stages, compared to wild-type embryos (Figs. 8B and E; 8C and F). The expression of pre-migratory NC markers was also severely reduced in the *arl6ip1*-MO1 embryos between the 3- and 8-somite stages (Fig. 5). We detected a great increase of TUNEL-positive cells around the dorsal ectoderm in *arl6ip1*-MO embryos from the 3- to 15-somite stages, and this increase ended at 36 hpf. To confirm whether these dying cells were overlapped with *crestin*-positive NC cells, we performed double- labeling with *crestin* and fluorescent TUNEL (Figs. 8G–I; G′–I′). The significant expression of *crestin* in wild-type embryos indicated that cranial NC appeared at the anterior region of trunk somites at this stage (Figs. 8G and G′, indicated by arrows). When we compared dying cells with *crestin*-positive NC cells using double-labeling assay in *arl6ip1*-MO1-injected embryos, apoptotic cells tended to localize at the more anterior and superficial region, while *crestin*-positive NC cells were inclined to localize at the posterior region (Figs. 8H and H′). Since cell death occurring in *arl6ip1*-MO1-injected embryos was inhibited by *p53-*MO, cell death was observed to be correspondingly decreased in the *arl6ip1*-MO1*/p53*-MO-injected embryos (Fig. 8I). Compared with wild-type embryos, we also found the number of *crestin*-positive cells in *arl6ip1*-MO1-injected embryos and *arl6ip1*-MO1*/p53*-MO-injected embryos to be reduced (Figs. 8G′ vs. 8H′; 8G′ vs. 8I′, indicated by brackets). In addition, most *crestin*-positive cells failed to show cell death signaling in *arl6ip1*-MO1-injected embryos (Figs. 8H and H′). To account for the overlapping signals of *crestin*- and TUNEL-positive cells under fluorescent stereomicroscopy, we found an average of 9.6, 6.7 and 1.9 out of 10 cells to be positive to both TUNEL and *crestin* signals in *arl6ip*-MO1-injected embryos, wild-type embryos, and *arl6ip1*-MO1*/p53*-MO- injected embryos, respectively. However, the appearance of TUNEL-positive cells did not correlate with most *crestin*-labeled cells at this stage, which could be attributed to other defects in the *arl6ip1* morphants. Thus, we concluded that cell death does not play a role in the NC survival of *arl6ip1*-MO1-injected embryos.

## Discussion

In this study, we are the first to investigate the novel function of Arl6ip1 in NC development of zebrafish. Specifically, by using *arl6ip1*-specific MO, we demonstrated that NC induction occurs normally, but Arl6ip1 is required for specification of sublineages within the NC. In the *arl6ip1*-deficient embryos, we found that the expressions of *dlx2* and *crestin* in three streams of cranial NC are almost absent, resulting in marked craniofacial defects. Moreover, these defects are not confined to the craniofacial region. Rather, vagal-derived NC cells also fail to populate the enteric nervous system of *arl6ip1*-deficient embryos. We also showed a significant delay in the onset of migration in the trunk, and we demonstrated that such delay causes defects in dorsal root ganglia and synthesis of pigment cells in the *arl6ip1* morphants. Finally, the defects of *arl6ip1* morphants could be restored by injecting wobble *arl6ip1* mRNA. However, the degree of rescue by increasing *arl6ip1* mRNA concentration alone is not as effective as injecting *arl6ip1* mRNA/*p53-*MO. While it was concluded that *arl6ip1*-MO1 might have some off-targeting effects in embryos, *arl6ip1*-MO1/*p53*-MO co-injection could prevent these nonspecific effects. Therefore, all MO experiments carried out in this study used *arl6ip1*-MO1/*p53*-MO co-injection, except those unaltered genes in *arl6ip1*-MO1 embryos. Most importantly, our results clearly demonstrated that Arl6ip1 plays a key functional role in NC migration and specification of sublineages, which points to the likelihood of its association with Hirschsprung's disease and with the aberrant craniofacial characteristics of BBS.

In mouse, *arl6ip1* is expressed in all hematopoietic cell lineages, and Arl6ip1 protein is localized to intracytoplasmic membranes [21]. By analysis of the cellular location of *arl6ip1* expression, Pettersson *et al.* (2000) [21] suggested that *arl6ip1* may play a role in protein transport, membrane trafficking, or cell signaling during hematopoietic maturation. Lui *et al.* (2003) [22] also demonstrated that ARMER/Arl6ip1 is a novel ER integral membrane protein which protects cells by inhibiting caspase-9 activity, thereby revealing a possible role for ARMER in cell survival. Interestingly, in this study, we demonstrated that zebrafish *arl6ip1* is expressed ubiquitously through 24 hpf and plays a critical role in NC migration and specification of derivatives, rather than hematogenesis. By loss-of-function studies, the results presented in this report strongly support the hypothesis that zebrafish Arl6ip1 performs key functions in NC development, including derivates specification and migration, but not induction. Since Arl6 is a major regulator involved in BBS, the implications for human disease become obvious, particularly as they relate to neuropathic disorders.

In a previous report, genetic studies showed that *foxd3* is expressed normally in the pre-migratory neural crest cells of the *sox10* (*colorless*) mutant [36], suggesting that *foxd3* may function as an upstream effector of *sox10*. This hypothesis is supported by Kelsh and Eisen (2000) [36], Elworthy *et al.* (2005) [37] and Stewart *et al.* (2006) [38], who demonstrated the effect of *foxd3* on the *sox10*-dependent genetic pathway, where *foxd3* mutants (*sym1* mutants) appeared to show a more severe neural crest phenotype than *sox10* mutants, with the exception of chromatophores. Therefore, based on the genetic analysis presented in this study, it is plausible to theorize that *arl6ip1* may regulate the specification of NC derivatives through activation of other pre-migratory NC-specific transcription factors, such as *sox10* or *foxd3*.

Another mutant found to affect *foxd3* function is *mosm188*. A complementation analysis with the *sym1/foxd3* mutant found that the two mutations affect the same locus. Sequencing the *foxd3* coding region in *mosm188* mutants did not display any nucleotide changes, suggesting that *mosm188* most likely impairs either *foxd3* mRNA expression or stability. Furthermore, since *mosm188* mutants express *foxd3* ectopically in somites or in gastrula, but not in NC cells, it is likely that the *mosm188* mutation knocks out an NC-specific regulatory element within the *foxd3* locus [39]. This mutant also showed the same depletion of NC derivatives as *sym1* mutation, including craniofacial cartilage, sympathetic neurons, dorsal root ganglia, enteric neurons, and pigment cells. Moreover, it was also reported that the *foxd3* gene might act to maintain the undifferentiated state of NC progenitors during embryogenesis. In the absence of Foxd3, it is thought that some NC cells prematurely differentiate, thus producing fewer progenitor cells and leading, in turn, to the normal development of fewer derivatives. Although Arl6ip1 may act upstream of Foxd3 and Sox10 (Fig. 6), knockdown of Arl6ip1 still reduces the number of both *sox10*- and *foxd3*-expressing NC progenitors. Thus, Arl6ip1 knockdown causes more serious developmental defects in the reduction, or absence, of NC derivatives than *sox10* and *foxd3* mutants.

Furthermore, since Snail family members can affect early NC development in other species [40], [41], [42], the severe reduction of *snai1b* expression in *arl6ip1* morphants in NC subpopulations may also contribute to the neuronal and glial defects. However, since *snai1b* function has not yet been uncovered in zebrafish, it remains to be elucidated whether the *snai1b* gene also differentially regulates NC specification.

We found that both abnormal trunk extension and somite defects were induced by *arl6ip1*-MO1. Thus, it is possible that *arl6ip1*-MO1-injected embryos undergo mispatterning of somitic regions, causing a significant loss of cells in these regions that would otherwise provide guidance or survival cues to the trunk migrating NC cells. These defects could also be indirectly affected by trunk neural development, such as cell migration. Furthermore, Robu *et al.* (2007) [26] stated that *p53* knockdown by itself did not induce any significant defects, as *p53* is not required for normal development in mammals or fish (see Figures 1B and 2B of Robu *et al.*, 2007). In the present study, *p53*-MO did not affect the efficacy of gene-specific MOs, as it did not interfere with the penetrance of gene-specific phenotypes. Our findings also clearly showed that head size and trunk extension were recovered in *arl6ip1-*MO1/*p53*-MO-injected embryos at 24 hpf. In addition, we also confirmed that *myod* was expressed normally, either in wild-type or *arl6ip1*-MO1/*p53*-MO-injected embryos, at 24 hpf (data not shown). Thus, we used either *arl6ip1* or *arl6ip1/p53* morphants with only mild defects impeding normal trunk development and co-tested with *myod* staining to analyze the migration of trunk NC. Finally, we demonstrated that trunk NC migration, either in *arl6ip1* or *arl6ip1/p53* morphants, is defective.

The regulation of cell death by Arl6ip1 may be a conserved function. Lui *et al.* (2003) [22] gave evidence of this essential role in cell survival by induced overexpression of human Arl6ip1 in cell line HT1080. Briefly, their work demonstrated that human Arl6ip1 plays a possible role in cell survival by inhibiting caspase-9 activity. Recent studies have shown that Snail family members also play a role in the survival of progenitor cell populations and are overexpressed in some human cancers [43], [44], [45]. In our studies, however, double-labeling with *crestin* and TUNEL in *arl6ip1*-MO1 embryos showed that just a few of the dying cells were *crestin*-positive. Thus, zebrafish Arl6ip may play a role in cell death, but not in NC survival.

Because trunk NC cells were localized between the dorsal surface of the neural keel and overlying ectoderm in the *arl6ip1*-MO1 embryos, defects in dorsal root ganglia clearly resulted from the inhibition of trunk NC migration. Similarly, reduced numbers of dorsal root ganglia are to be found in the zebrafish *sym1* mutant. Stewart *et al.* (2006) [38] demonstrated that *foxd3* functions to specify neuronal and glial sublineages and is required for the timing of trunk NC migration. Furthermore, decreased expression of *sox10*, *snai1b* and *ctn* was evident in *sym1* mutants. It is worthwhile noting that down-regulation of early NC specifiers may also affect the migration of the NC cells at the later embryonic stage. This result is also consistent with recent studies in chick showing that *foxd3* function is required for NC migration by regulating the expression of cell-cell adhesion molecules [46], [47], [48]. Therefore, the results of our zebrafish study are in agreement with those findings in chick and *sym1* mutants since we found that *foxd3* retains sufficient expression in the somite of zebrafish embryos at 30 hpf to inhibit trunk NC migration (data not shown).

The early NC phenotype reported here is severe at the 3-somite stage (Fig. 5), yet the NC phenotype reported later in development (Fig. 6) appears much less severe. We do not think that the NC phenotypes, which are much less severe in later development, as shown in Figure 6, are a consequence of developmental delay. All the NC sublineages are derived from the pre-migratory cell pool at the 3-somite stage, but no clear region can be found indicating localization of the pool of pre-migratory cells that might contribute to the subset of NC sublineages. However, based on the defective NC derivatives which occurred in the *arl6ip1*-MO1-injected embryos, we learned which NC sublineages are affected. Consequently, in this study, we hypothesized that the subset of NC sublineages of trunk was less affected by the knockdown of Arl6ip1 in the pre-migratory NC pool. In contrast, the subsets of vagal and cranial NC cells were greatly reduced in the pre-migratory NC pool of *arl6ip1*-MO1-injected embryos. Therefore, at the later developmental stages, development of both vagal-derived enteric neurons and cranial-derived pharyngeal arches was highly reduced in cell numbers. On the other hand, the trunk NC cells appeared much less affected in terms of cell number. Knecht and Bronner-Fraser (2002) [49] reported that Wnt, Bmp2/4 and TGFβ 1, 2 and 3 signaling pathways induce multipotent NC progenitor cells and that the newly specified pre-migratory NC progenitors express a unique set of transcription factors, such as Foxd3, Snai1b, Id2, Sox10 and Tcfap2a. Subsequently, the NC cells turn off early markers, like Sox10, up-regulate a new set of genes, and begin to migrate. At this stage, NC cells derived from the hindbrain migrate as a wave in a caudal direction. The first group of cells starting to migrate includes the cells populating in the pharyngeal arches. This first group of cells is followed by a second wave of migration which supplies cells to trunk NC [50]. In Figures 5F–H, we can clearly observe that the pre-migratory NC of midbrain-hindbrain boundary in the *arl6ip1*-MO1-injected embryos was only slightly affected. On the other hand, the trunk NC cells could not migrate ventrally in the *arl6ip1*-MO1-injected embryos. However, the proliferation of trunk NC cells may be normal, resulting in non-migrated trunk NC cells populating the dorsal neural tube. This continuous proliferation of trunk NC cells recovers the loss of the NC cells in the early developmental stages. Actually, the subsets of vagal and cranial NC cells do not recover at all. These findings might plausibly explain why the trunk NC cell numbers were less severe.

*Arl6* is the gene underlying BBS type 3 [23]. Analysis of BBS morphants in zebrafish showed that aberrant neural crest migration underlies the craniofacial and enteric nervous system defects mirroring mammalian mutants [24], suggesting that BBS-associated genes might be involved in NC migration. In our studies, *arl6ip1* morphants exhibit similar defective phenotypes, such as craniofacial dysmorphology and inhibition of trunk NC migration. We also found the loss of enteric neurons in *arl6ip1* morphants. Therefore, we concluded that Arl6ip1 might function in NC cell development through interaction with Arl6. In addition, by loss-of-function studies, our results showed that knockdown of *arl6ip1* negatively affects NC development, indicating, in turn, that *arl6ip1* could be an underlying etiological factor in the onset of BBS and other neurocristopathies.

## Materials and Methods

### Fish husbandry and observation

The wild-type AB strain of zebrafish was cultured at 28.5°C. Embryos were staged by hpf [51]. The phenotype of heart formation was observed under a fluorescent stereomicroscope, MZ FLIII (Leica). Images were captured with a Fine pix S2 pro camera (Nikon) using the Camera Shooting software.

### MO knockdown

The MOs were specifically designed to inhibit the translation of *arl6ip1* mRNA as follows: fluorescence-tagged *arl6ip1*-MO1 and *arl6ip1*-MO1, 5′-ACTTTTATTGT- CGCCCTCAGCCATG-3′, and *arl6ip1*-MO2, 5′-GATGTTACTTGAGAGTTTAGG- TTCC-3′. A control MO specific for *Arl6ip1* was designed as 5′-GTACCGACT- CCCGCTGTTATTTTCA-3′, which is an inverted sequence of *arl6ip1*-MO1. We also designed *p53*-MO, 5′-GCGCCATTGCTTTGCAAGAATTG-3′, to suppress the cell death induced by MO off-targeting. The amount of *p53-*MO injected was 1.5-fold (w/w) higher than the amount of MO previously described [26]. All MOs were prepared at a stock concentration of 1 mM and diluted to the desired concentration for microinjection into each embryo.

### Whole-mount *in situ* hybridization and TUNEL assay

Digoxigenin (DIG)-labeled antisense RNA probe used for whole-mount *in situ* hybridization was generated by using DIG RNA labeling kit (Roche). Embryos at the desired stage were collected and then fixed with 4% paraformaldehyde for 4 h at 25°C. After fixation, embryos were dechrionized, dehydrated in absolute alcohol, and stored at −20°C. Embryos were rehydrated by immersion for 10 min each in 75%, 50%, and 25% ethanol. Rehydrated embryos were transferred to phosphate buffer saline (PBS). Prehybridization, hybridization, and detection procedures were performed with a DIG detection kit II, according to the instructions in the supplier's manual (Boehriner Mannheim). The TUNEL assay was described previously [52] using The DeadEnd™ Colorimetric TUNEL System (Promega) or DeadEnd™ Fluorometric TUNEL System. The overlapping signals of *crestin*- and fluorescent TUNEL-positive cells were detected and counted under a fluorescent stereomicroscope, MZ FLIII (Leica).

### Plasmid construction

In order to further demonstrate the specificity of MO targeting, we constructed plasmids of pCS2-Arl6ip1-GFP, in which the binding sequence of *arl6ip1*-MO1 was fused with the GFP reporter cDNA. The *Not*I-cut pCS2-Arl6ip1-GFP served as a template to synthesize RNA using the mMessage Machine kit (Ambion). The resultant *arl6ip1*-MO1-target-*gfp* RNA was injected at 100 pg per embryo or co-injected with 4 ng *arl6ip1*-MO1 into one-celled zebrafish embryos. Appearance of the GFP signal in the treated embryos was observed at the 24-hpf stage using green fluorescent microscopy.

### Rescue experiment

Since the introduced *arl6ip1* mRNA was not bound by *arl6ip1*-MO1 during the rescue experiment, we designed a wobble *arl6ip1* and *arl6ip1-gfp* mRNA. The wobble *arl6ip1*-*gfp* mRNA was a wobble *arl6ip1* cDNA fused in frame with *egfp* cDNA, and we changed the nt 125–149 of zebrafish *arl6ip1* cDNA (GenBank Accession NO. NM_201112) from 5′-ATGGCTGAGGGCGACAATAAAAGTG-3′ to 5′-ATGGCcGAaGGaGAtAAcAAgAGcG-3′, but without altering the amino acid residues. Capped mRNA transcripts of wobble *arl6ip1* and wobble *arl6ip1*-gfp for rescue experiments were synthesized by SP6 *in vitro* transcription, according to the protocol of the manufacturer (Epicentre). The resultant mRNAs were diluted to the desired concentration (from 100 to 300 pg/µl), and approximately 2.3 nl were microinjected into one-celled stage embryos.

### Whole-mount immunofluorescence staining

Anti-Hu antibodies were used to detect cranial ganglia, enteric neurons, and dorsal root ganglia, as described by O'Brien *et al.* (2004) [53]. After embryos were fixed in 0.4% paraformaldehyde at pH 7.2 for 4 hr at room temperature, they were washed three times with PBS for 15 min each, soaked in 100% acetone at −20°C for at least 10 min, rehydrated through graded methanol in PBS and placed in blocking solution at room temperature for 1 hr (5% sheep serum, 1% bovine serum albumin (BSA), 1% dimethylsulfoxide and 0.1% Tween 20 in PBS). After blocking, the embryos were subjected to primary antibody at 4°C overnight. The embryos were then extensively washed with 1% BSA in PBST and incubated at 4°C overnight with gentle mixing in goat anti-mouse immunoglobulin conjugated with Cy3, 2 mg/ml diluted 1∶50 in blocking solution. The embryos were washed extensively in 1% BSA in PBST and finally in PBS.

### Cartilage staining

The Alcian blue staining protocol followed Neuhauss *et al.* (1996) [54] with some modifications. Embryos at 4 dpf were anesthetized using 0.02% buffered tricaine (Sigma) and fixed overnight in 4% PFA at 4°C. After washing with PBS, embryos were stained overnight in 0.1% Alcian blue, which was dissolved in acidic ethanol (70% ethanol, 5% concentrated hydrochloric acid), then washed extensively in acidic ethanol, dehydrated, and stored in 80% glycerol. For better exposure of cartilage elements, embryos were digested with 0.02% trypsin.

## Supporting Information

Figure S1 **Confirmation of specific activities of** ***arl6ip1*** **-MO1 in zebrafish embryos.** (A) Injection of 4 ng *arl6ip1*-MO1 caused brain lesions and foreshortened trunks. The defects of *arl6ip1*-MO1 were rescued either partially by the wobble *arl6ip1* mRNA (E) and wobble *arl6ip1*-*gfp* mRNA (B) or almost completely by wobble *arl6ip1*-*gfp* mRNA with *p-53* MO (C). (F, G) By detecting the GFP signals, we confirmed that the *arl6ip1*-MO1 cannot target wobble *arl6ip1*-*gfp* mRNA. (D) The Arl6ip1-GFP fusion protein was detected at 24 hpf in embryos injected with *arl6ip1*-MO1-target*-gfp* mRNA. (H) The GFP signal was absent at 24 hpf in embryos co-injected with arl6ip1-MO1-target*-gfp* mRNA and *arl6ip1*-MO1. (I) The *arl6ip1* morphants did not display sulcus and gyrus in brains, and these brain defects were rescued either partially by wobble *arl6ip1*-*gfp* mRNA (J) or completely by wobble *arl6ip1*-*gfp* mRNA with *p53*-MO (K).
(TIF)

Figure S2 **The** ***arl6ip1*** **-MO1-injected embryos do not appear to have defective patterning in hindbrain.** Wild-type (WT; A, C, E) and *arl6ip1-*knockdown (MO; B, D, F) embryos, either at 3-somite stage (3ss) (A–D) or at 24 hpf (E, F), were observed at dorsal view. (A–D) Neither *fgf3* expression nor *fgf8* expression in the *arl6ip1* morphants was distinguishable from that of wild-type embryos (A vs. B; C vs. D). (E, F) Dorsal views of 24 hpf embryos processed for anti-Hu immunofluorescence staining (IFA) to reveal hindbrain segmentation. Similar to WT embryos, *arl6ip1-*knockdown embryos showed normal r1-r7 segmentation. fb, forebrain; mhb, midbrain-hindbrain border; pr4, premature rhombomere 4; r1–r7, rhombomere 1–7.
(TIF)

Figure S3 **Induction of neural crest cells occurs normally in** ***arl6ip1*** **-MO1 embryos.** (A, B) Dorsal views of embryos at 10 hpf processed to show *sox2* expression (labeling neural plate: np), and *dlx3b* expression (labeling the pre-placodal region: ppr). (A′, B′) Higher magnification views of right side of embryos shown in panels A and B, respectively. The region between these two expression domains was normally occupied by pre-migratory neural crest cells (indicated by brackets in A′ and B′).
(TIF)

Figure S4 **Injection of 75 pg wobble** ***arl6ip1*** **mRNA enables embryos to rescue the defects induced by 4 ng** ***arl6ip1*** **-MO1.** (A–C) Dorsal views of 3-somite-stage embryos, anterior to the top. (D–F) Lateral views of 24 hpf embryos, anterior to the left. (A, B) Compared to wild-type embryos, decreased expression of *foxd3* was evident and predominant, especially in caudal region of pre-migratory neural crest cells in *arl6ip1* morphants. (C) The down-regulation of *foxd3* in *arl6ip1*-MO1 embryos was rescued by the wobble *arl6ip1* mRNA. (D, E) *Crestin*-positive neural crest cells that normally migrate ventrally from the neural tube into the trunk were disrupted in *arl6ip1* morphants (indicated by arrows). (F) Co-injection of wobble *arl6ip1* mRNA with *arl6ip1*-MO1 recovered neural crest migration. (G) Quantification of the number of streams of *sox10*-labeled neural crest cells certified that migratory defects in *arl6ip1* morphants can be rescued by wobble *arl6ip1* mRNA.
(TIF)

Figure S5 Abnormal Sonic Hedgehog signaling in *arl6ip1*-MO1*/p53*-MO morphants. (A–D) Lateral views of 24hpf embryos, anterior to the left. (A, B) Expression of *shh* was normal, either in wild-type embryos (A) or *arl6ip1*-MO1*/p53*-MO-injected embryos (B). (C, D) *pax6*, a gene negatively regulated by Shh signaling, was up-regulated in the neural tube of *arl6ip1*-MO1*/p53*-MO-injected embryos (D). Neural tube: indicated by arrow.
(TIF)

Figure S6 Knockdown of Arl6ip1 induces KV cilia defects. (A–D) *pitx2* normal expression in left lateral plate mesoderm (LPM)(arrow) at 20-somite stage was disrupted in embryos injected with *arl6ip1*-MO1*/p53*-MO. (E–H) At 20-somite stage, *spaw* is expressed in the left LPM (arrow), while knockdown of Arl6ip1/P53 resulted in left (E), absent (F), bilateral (G) or rightward (H) *spaw* expression in the LPM. (I, J) Confocal microscopic images of anti-acetylated tubulin staining of KV cilia (red fluorescent) at 8-somite stage. In the wild-type embryos, cilia can be observed in a spherical pattern in the region of KV (I). Cilia number, length and KV area reduced in *arl6ip1*-MO1*/p53*-MO morphants at 8-somite stage (J). (K) Summary of *pitx2* and *spaw* asymmetrical gene expression patterns.
(TIF)

Figure S7 Specific knockdown of Arl6ip1 in DFCs alters LR development without other effects on embryogenesis. (A) As observed under fluorescent microscopy, injection of fluorescein-tagged *arl6ip1*-MO1 into mid-blastula stage embryos was observed in dorsal forerunner cells (DFCs) 3–4 hr post-injection at 70% epiboly stage. The dorsal margin was indicated by dashed line. (B–E) Lateral views of 24hpf embryos. Embryos were observed under transmitted light microscopy (B, C) or fluorescent microscopy (D, E). MOs were present in all cells of embryos injected at the one-cell stage (D), and severe defects induced by fluorescein-tagged *arl6ip1*-MO1 embryos were shown (B). Fluorescein-tagged *arl6ip*1-MO1 injected into embryos at mid-blastula stage was primarily found in the yolk cell and yolk tube (E), indicating that cells, other than DFC and yolk, did not incorporate MO. These DFC of *arl6ip1*-MO1-injected embryos (DFC*^arl6ip1^* ^-MO1^) developed a normal morphology (C) similar to WT embryos. (F, G) Tail views of 6-somite stage embryos. KV was indicated by arrows. Compared to WT embryos (F), DFC*^arl6ip1^* ^-MO1^ embryos showed reduced KV at 6-somite stage (G). (H, I) Confocal microscopy images of anti-acetylated tubulin staining of KV cilia (red fluorescent) at 6-somite stage. Cilia distributed over a spherical pattern in the region of KV (H). However, cilia were disorganized in reduced KV of DFC*^arl6ip1^* ^-MO1^ embryos (I). (K) Summary of *pitx2* and *spaw* asymmetrical gene expression patterns.
(TIF)
