# Supplementary figures and images for: Zebrafish arl6ip1 Is Required for Neural Crest Development during Embryogenesis
Source: PLoS One. 2012 Mar 9;7(3):e32899. doi: 10.1371/journal.pone.0032899 (PMC3298456; doi:10.1371/journal.pone.0032899)

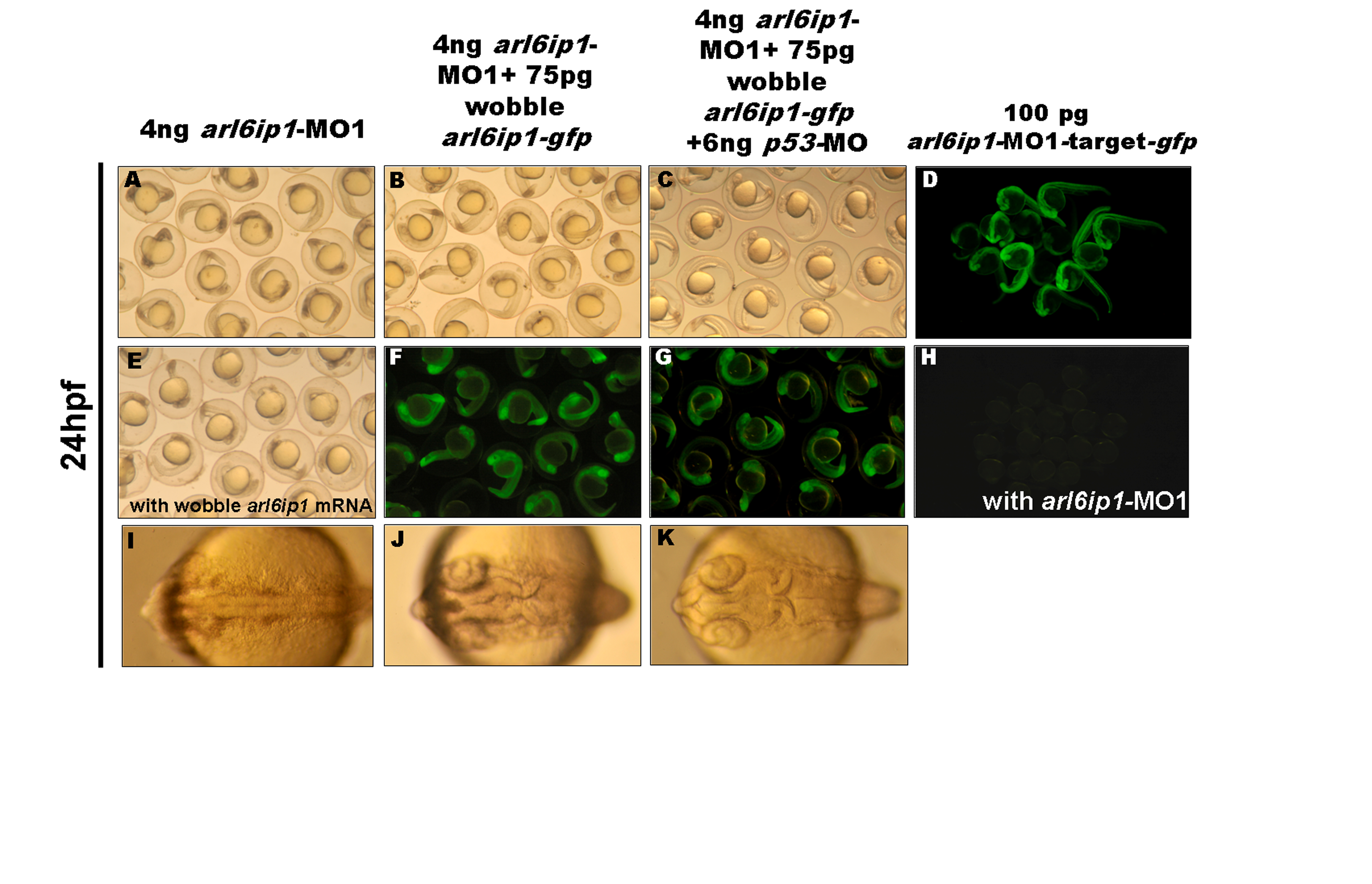

Supplement: Figure S1 — Confirmation of specific activities of arl6ip1 -MO1 in zebrafish embryos. (A) Injection of 4 ng arl6ip1-MO1 caused brain lesions and foreshortened trunks. The defects of arl6ip1-MO1 were rescued either partially by the wobble arl6ip1 mRNA (E) and wobble arl6ip1-gfp mRNA (B) or almost completely by wobble arl6ip1-gfp mRNA with p-53 MO (C). (F, G) By detecting the GFP signals, we confirmed that the arl6ip1-MO1 cannot target wobble arl6ip1-gfp mRNA. (D) The Arl6ip1-GFP fusion protein was detected at 24 hpf in embryos injected with arl6ip1-MO1-target-gfp mRNA. (H) The GFP signal was absent at 24 hpf in embryos co-injected with arl6ip1-MO1-target-gfp mRNA and arl6ip1-MO1. (I) The arl6ip1 morphants did not display sulcus and gyrus in brains, and these brain defects were rescued either partially by wobble arl6ip1-gfp mRNA (J) or completely by wobble arl6ip1-gfp mRNA with p53-MO (K). (TIF) [file pone.0032899.s001.tif]

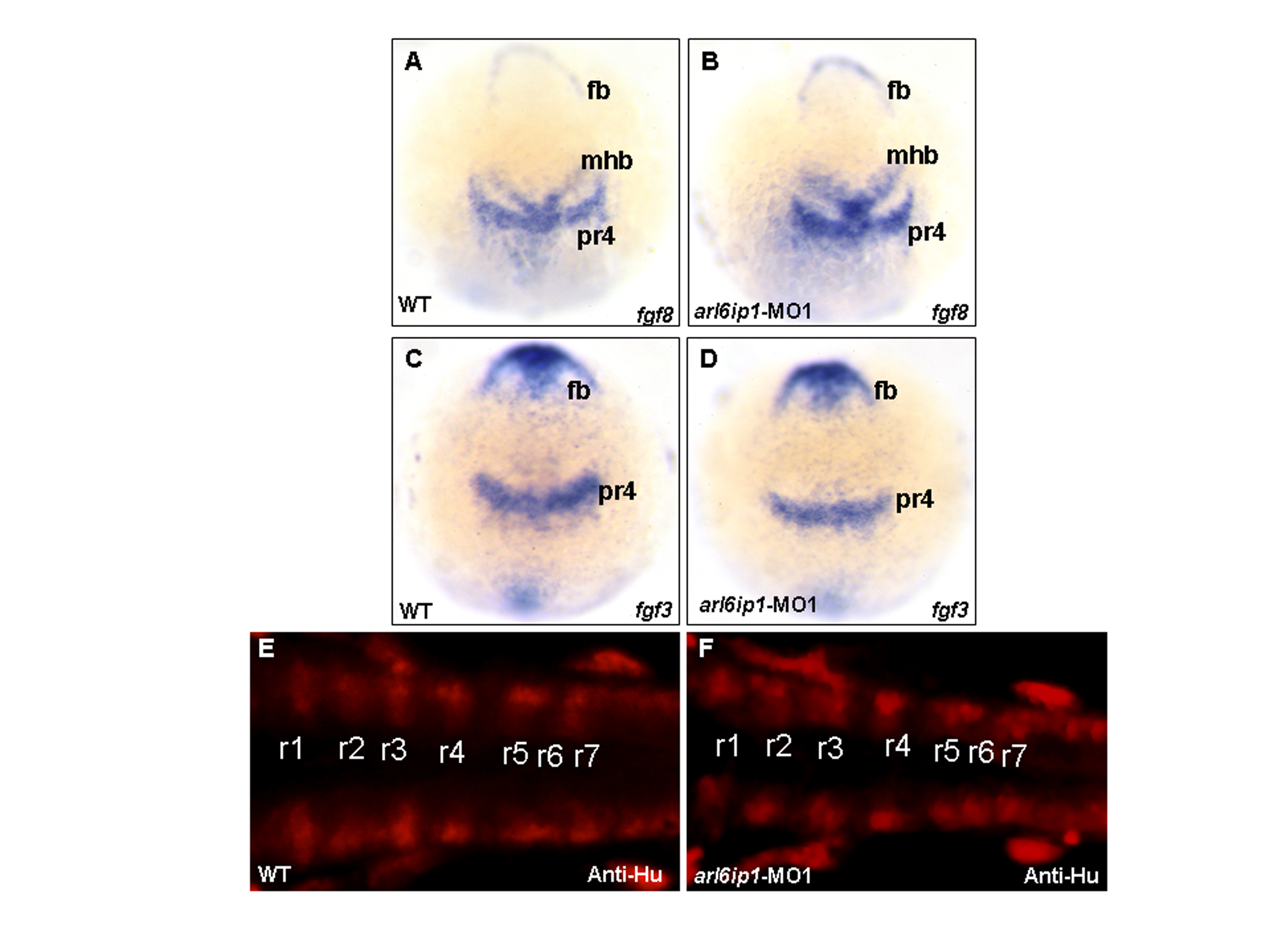

Supplement: Figure S2 — The arl6ip1 -MO1-injected embryos do not appear to have defective patterning in hindbrain. Wild-type (WT; A, C, E) and arl6ip1-knockdown (MO; B, D, F) embryos, either at 3-somite stage (3ss) (A–D) or at 24 hpf (E, F), were observed at dorsal view. (A–D) Neither fgf3 expression nor fgf8 expression in the arl6ip1 morphants was distinguishable from that of wild-type embryos (A vs. B; C vs. D). (E, F) Dorsal views of 24 hpf embryos processed for anti-Hu immunofluorescence staining (IFA) to reveal hindbrain segmentation. Similar to WT embryos, arl6ip1-knockdown embryos showed normal r1-r7 segmentation. fb, forebrain; mhb, midbrain-hindbrain border; pr4, premature rhombomere 4; r1–r7, rhombomere 1–7. (TIF) [file pone.0032899.s002.tif]

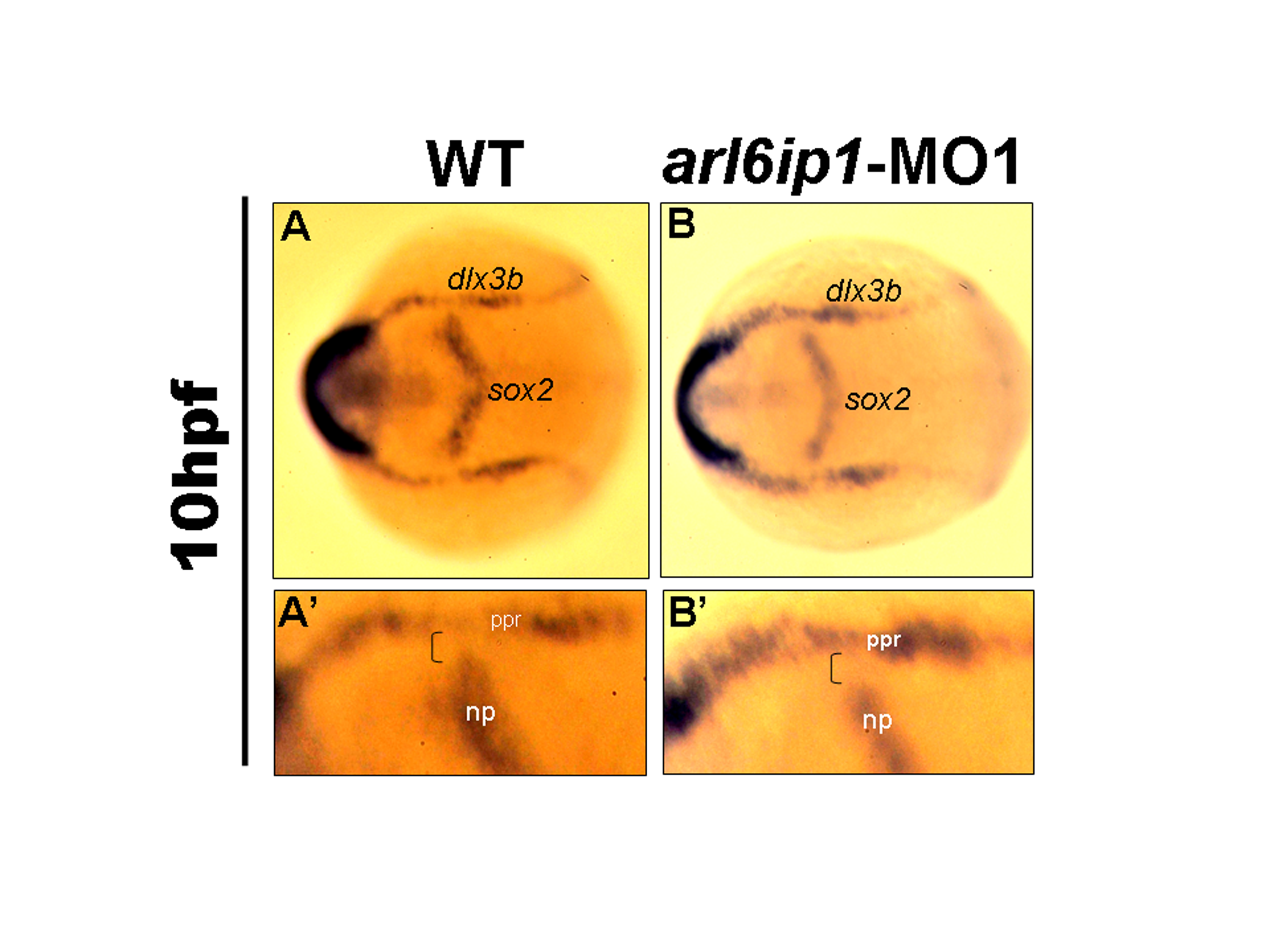

Supplement: Figure S3 — Induction of neural crest cells occurs normally in arl6ip1 -MO1 embryos. (A, B) Dorsal views of embryos at 10 hpf processed to show sox2 expression (labeling neural plate: np), and dlx3b expression (labeling the pre-placodal region: ppr). (A′, B′) Higher magnification views of right side of embryos shown in panels A and B, respectively. The region between these two expression domains was normally occupied by pre-migratory neural crest cells (indicated by brackets in A′ and B′). (TIF) [file pone.0032899.s003.tif]

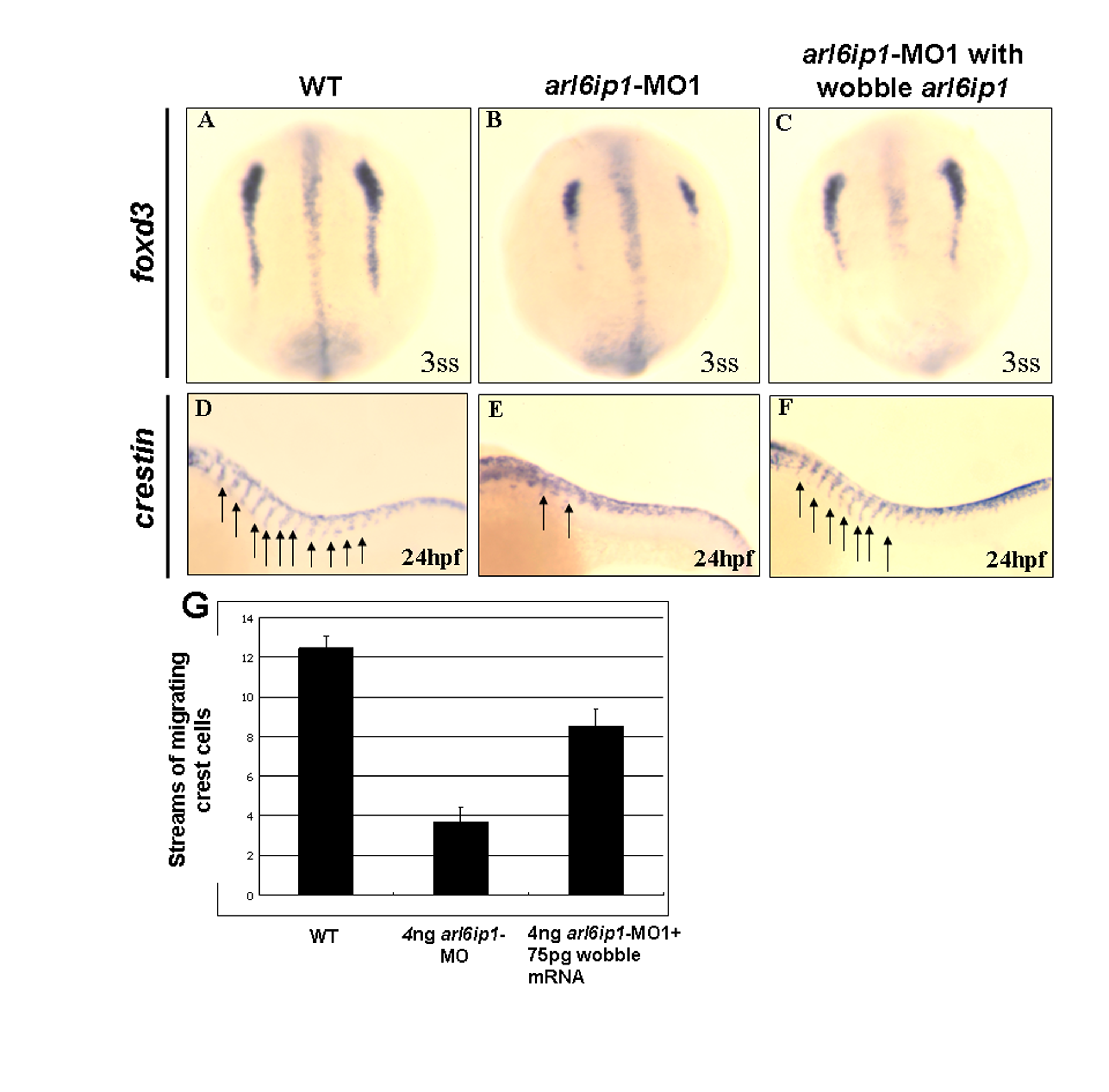

Supplement: Figure S4 — Injection of 75 pg wobble arl6ip1 mRNA enables embryos to rescue the defects induced by 4 ng arl6ip1 -MO1. (A–C) Dorsal views of 3-somite-stage embryos, anterior to the top. (D–F) Lateral views of 24 hpf embryos, anterior to the left. (A, B) Compared to wild-type embryos, decreased expression of foxd3 was evident and predominant, especially in caudal region of pre-migratory neural crest cells in arl6ip1 morphants. (C) The down-regulation of foxd3 in arl6ip1-MO1 embryos was rescued by the wobble arl6ip1 mRNA. (D, E) Crestin-positive neural crest cells that normally migrate ventrally from the neural tube into the trunk were disrupted in arl6ip1 morphants (indicated by arrows). (F) Co-injection of wobble arl6ip1 mRNA with arl6ip1-MO1 recovered neural crest migration. (G) Quantification of the number of streams of sox10-labeled neural crest cells certified that migratory defects in arl6ip1 morphants can be rescued by wobble arl6ip1 mRNA. (TIF) [file pone.0032899.s004.tif]

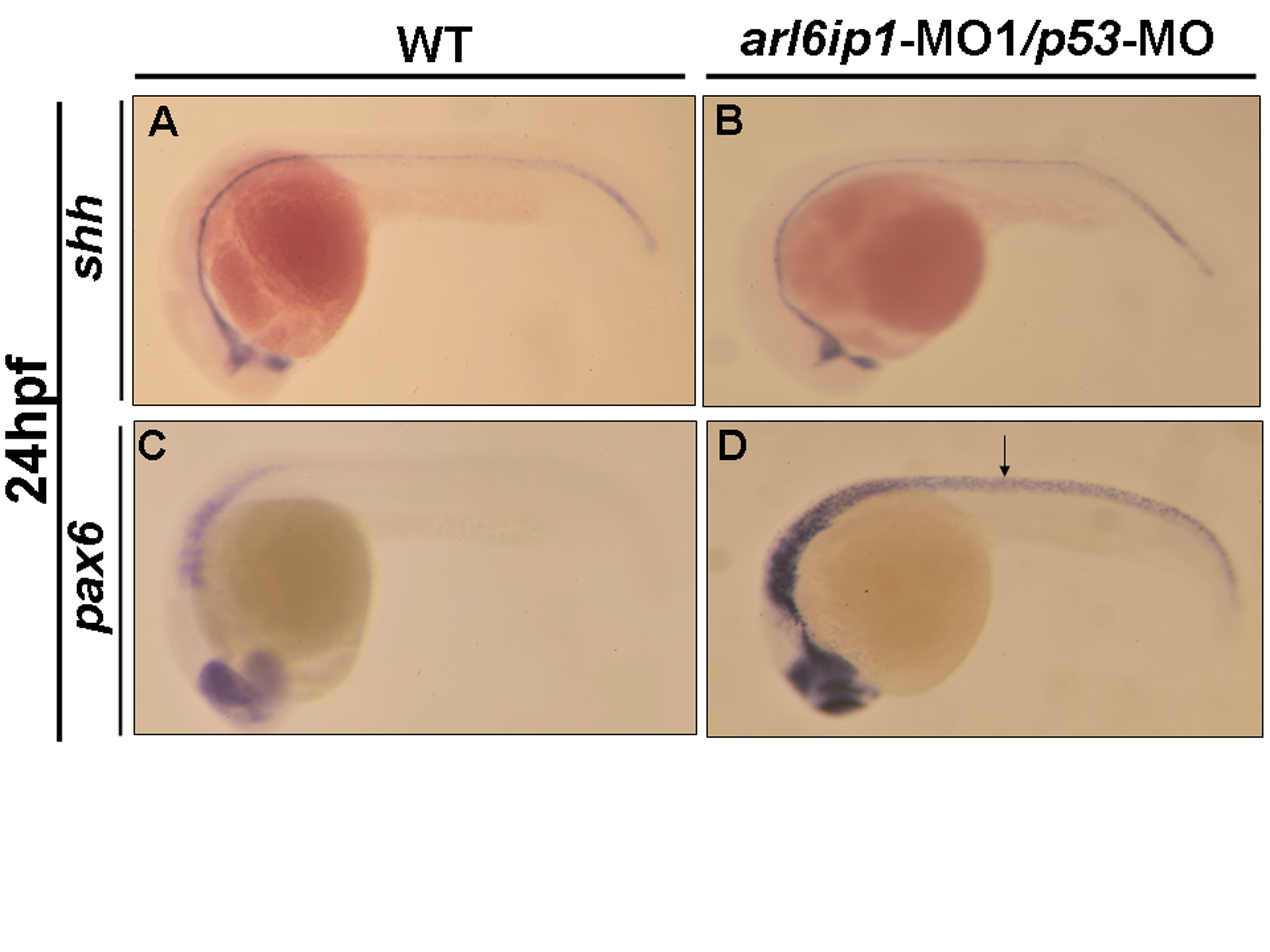

Supplement: Figure S5 — Abnormal Sonic Hedgehog signaling in arl6ip1-MO1/p53-MO morphants. (A–D) Lateral views of 24hpf embryos, anterior to the left. (A, B) Expression of shh was normal, either in wild-type embryos (A) or arl6ip1-MO1/p53-MO-injected embryos (B). (C, D) pax6, a gene negatively regulated by Shh signaling, was up-regulated in the neural tube of arl6ip1-MO1/p53-MO-injected embryos (D). Neural tube: indicated by arrow. (TIF) [file pone.0032899.s005.tif]

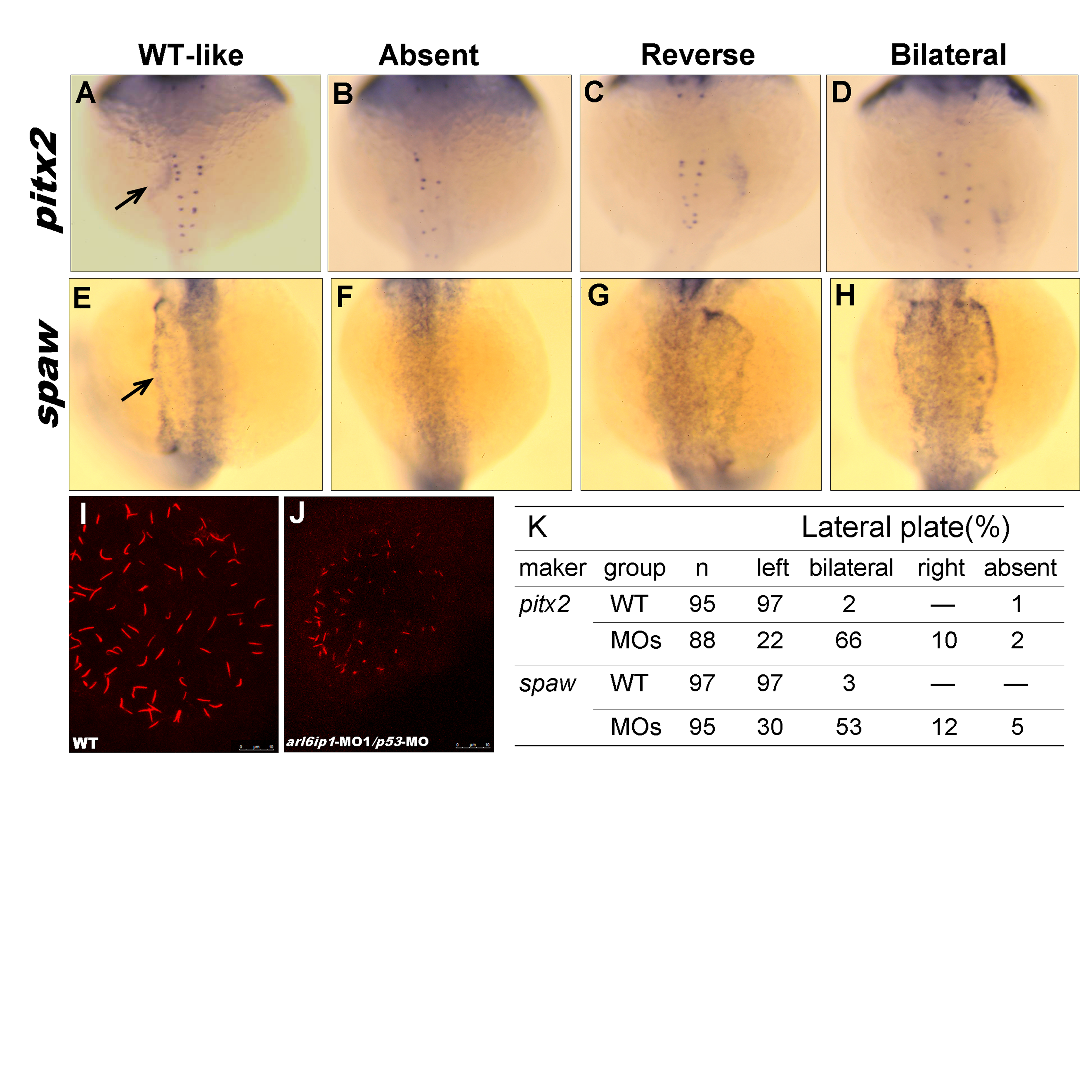

Supplement: Figure S6 — Knockdown of Arl6ip1 induces KV cilia defects. (A–D) pitx2 normal expression in left lateral plate mesoderm (LPM)(arrow) at 20-somite stage was disrupted in embryos injected with arl6ip1-MO1/p53-MO. (E–H) At 20-somite stage, spaw is expressed in the left LPM (arrow), while knockdown of Arl6ip1/P53 resulted in left (E), absent (F), bilateral (G) or rightward (H) spaw expression in the LPM. (I, J) Confocal microscopic images of anti-acetylated tubulin staining of KV cilia (red fluorescent) at 8-somite stage. In the wild-type embryos, cilia can be observed in a spherical pattern in the region of KV (I). Cilia number, length and KV area reduced in arl6ip1-MO1/p53-MO morphants at 8-somite stage (J). (K) Summary of pitx2 and spaw asymmetrical gene expression patterns. (TIF) [file pone.0032899.s006.tif]

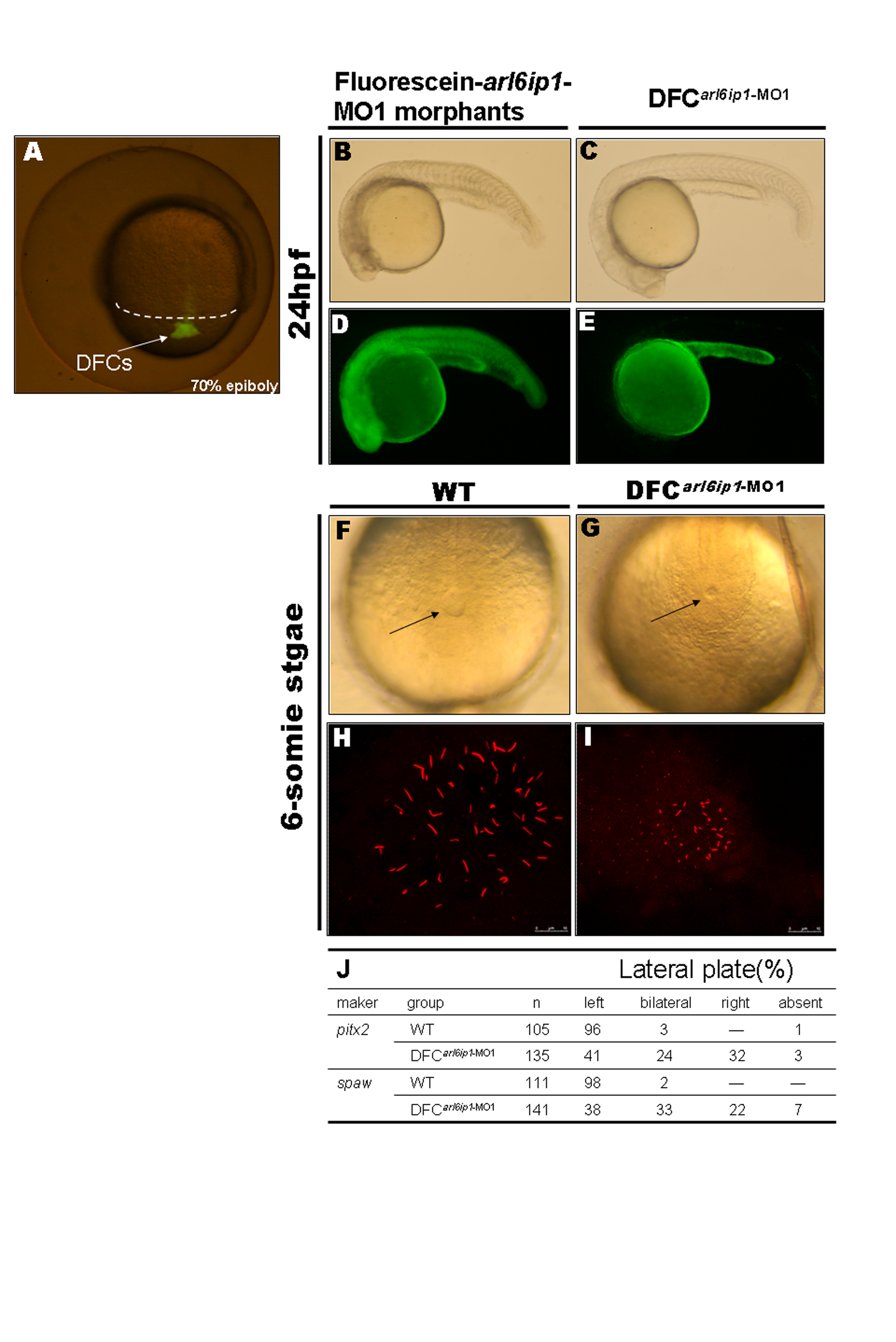

Supplement: Figure S7 — Specific knockdown of Arl6ip1 in DFCs alters LR development without other effects on embryogenesis. (A) As observed under fluorescent microscopy, injection of fluorescein-tagged arl6ip1-MO1 into mid-blastula stage embryos was observed in dorsal forerunner cells (DFCs) 3–4 hr post-injection at 70% epiboly stage. The dorsal margin was indicated by dashed line. (B–E) Lateral views of 24hpf embryos. Embryos were observed under transmitted light microscopy (B, C) or fluorescent microscopy (D, E). MOs were present in all cells of embryos injected at the one-cell stage (D), and severe defects induced by fluorescein-tagged arl6ip1-MO1 embryos were shown (B). Fluorescein-tagged arl6ip1-MO1 injected into embryos at mid-blastula stage was primarily found in the yolk cell and yolk tube (E), indicating that cells, other than DFC and yolk, did not incorporate MO. These DFC of arl6ip1-MO1-injected embryos (DFCarl6ip1 -MO1) developed a normal morphology (C) similar to WT embryos. (F, G) Tail views of 6-somite stage embryos. KV was indicated by arrows. Compared to WT embryos (F), DFCarl6ip1 -MO1 embryos showed reduced KV at 6-somite stage (G). (H, I) Confocal microscopy images of anti-acetylated tubulin staining of KV cilia (red fluorescent) at 6-somite stage. Cilia distributed over a spherical pattern in the region of KV (H). However, cilia were disorganized in reduced KV of DFCarl6ip1 -MO1 embryos (I). (K) Summary of pitx2 and spaw asymmetrical gene expression patterns. (TIF) [file pone.0032899.s007.tif]
